# Targeting MEF2A suppresses microglial hyperactivation and synaptic phagocytosis to attenuate epilepsy pathogenesis

**DOI:** 10.1038/s41419-026-08860-5

**Published:** 2026-05-22

**Authors:** Jingheng Wu, Jiayuanyuan Fu, Shuai Wang, Yuzhang Wu, Xu Wang, Shangang Feng, Qi Shi, Yuhao Wang, Yetong Shi, Yehong Fang, Yu Lan, Qiaoli Wu, Chuan Du, Shaoya Yin, Lixia Xu, Hua Yan

**Affiliations:** 1 Huanhu Hospital Affiliated to Tianjin Medical University, Tianjin Medical University, Tianjin, China; 2 Tianjin Key Laboratory of Cerebral Blood Flow Reconstruction and Head and Neck Tumor New Technology Translation, Tianjin Neurosurgical Institute, Tianjin Huanhu Hospital, Tianjin, China; 3 Department of Functional Neurosurgery, Shengjing Hospital of China Medical University, Shenyang, China

**Keywords:** Epilepsy, Epilepsy

## Abstract

Microglia’s role in epilepsy through neuroimmune communication is poorly understood. Mechanisms by which neurons activate microglia and how microglia affect neuronal activity to drive seizure-related inflammation remain unclear. Here, we elucidated a crucial axis connecting pathological adenosine triphosphate (ATP) release induced by epileptiform neuronal activity to microglial MEF2A-dependent hyperactivation, which exacerbates epilepsy pathology. In epilepsy models, seizures cause excessive ATP release, activating microglial P2X7 receptors, causing CAMKII phosphorylation. This triggers HDAC5 translocation, freeing MEF2A for acetylation, and enhancing transcription. Acetylated MEF2A increases CD74 and NEK7 expressions, enhancing NLRP3 inflammasome activation and microglial hyperactivation, worsening neuronal hyperexcitability by increasing inhibitory synapses clearance. Targeting microglial MEF2A with parecoxib or AAV knockdown reduced seizure severity and cognitive deficits and maintained synaptic inhibition by reducing excessive microglial phagocytosis. This reveals an ATP-P2X7-Ca^2^⁺- MEF2A signaling axis connecting neuronal injury with pathogenic microglial activation, suggesting MEF2A as a therapeutic target for microglial-neuronal homeostasis restoration in epilepsy pathology.

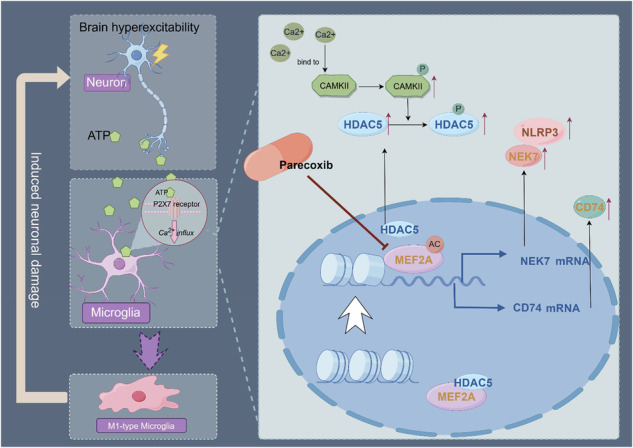

## Introduction

Epilepsy, a chronic neurological disorder characterized by spontaneous and recurrent seizures, affects 50–70 million people globally [1, 2]. Its etiologies, pathogenesis, clinical manifestations, and outcomes vary significantly, with unknown mechanisms underlying seizure onset and recurrence occurring in approximately 60% of cases. Despite the availability of various antiepileptic drugs (AEDs) targeting seizure activity, one-third of patients experience drug-resistant epilepsy [3, 4]. Current AEDs suppress abnormal neuronal electrical activity [5]. Nonetheless, recent advancements are focused on the microglia to regulate epileptogenesis [6–8]. Microglia actively modulate neuronal excitability and synaptic plasticity by monitoring neuronal activity, yet contribute to neuronal damage through neuroinflammatory pathways [9–12]. Elucidating interactions between microglial activity and epileptogenesis is essential to identifying therapeutic targets and addressing current AED limitations.

Merely depleting microglia or directly inhibiting their signaling receptors is insufficient for effective epilepsy treatment [13, 14]. Rather, this may exacerbate seizure severity and initiate subsequent damages. This suggests that microglia’s role in epilepsy extends beyond simple inflammatory responses. Abnormal microglia phagocytic activity relates to neuronal and synaptic loss, and spontaneous recurrent epileptic seizures [15, 16]. Specifically, excessive pruning of inhibitory synapses by microglia can disrupt excitatory-inhibitory balance, leading to neuronal hyperexcitability, which is a hallmark of epilepsy [17]. Consequently, therapeutic strategies targeting microglia may necessitate more precise modulation to circumvent the adverse effects of straightforward depletion or inhibition.

Considering microglial function complexity and targeted intervention necessity, adenosine triphosphate (ATP) was identified as crucial for epileptiform activity [18]. However, ATP can also trigger harmful effects via P2X7 receptor activation, leading to excessive phagocytosis and inflammation, exacerbating neuronal damage [19–26]. Understanding the mechanisms regulating ATP’s dual roles would be key to developing targeted therapies inhibiting pathogenic pathways while preserving neuroprotective functions.

ATP signaling orchestrates microglial activation through P2X and P2Y receptors, facilitating migration, cytokine secretion, and inflammasome activation processes [27–29]. This is associated with transcriptional regulation, wherein transcription factors act as pivotal intermediaries connecting extracellular signals to alter gene expression [30, 31]. Myocyte enhancer factor (MEF) transcription factors are crucial for regulating microglial responses. MEF2C, for example, curbs excessive microglial activation by inhibiting CDK2 kinase, to maintain neuroimmune balance [32]. Its extensive role in nervous system biology also manifests via association with genes vital for neuronal development and mitochondrial function [33]. MEF2D also directly influences microglial inflammatory responses [34]. MEF2A’s unique expression in microglia indicates its potential role as a key neuroimmune response regulator [35]. However, the specific molecular mechanism by which MEF2A influences ATP-driven microglial activation is poorly understood, prompting further research into its therapeutic potential.

Here, we investigated the function of microglial MEF2A as a pivotal integrator of ATP-mediated signaling and neuroimmune dysregulation in the acute phase of epilepsy, through combined in vivo, in vitro, and clinical analyses.

## Results

### MEF2A drives microglial activation through spatiotemporal-specific expression in epileptic progression

**Fig. 1:**
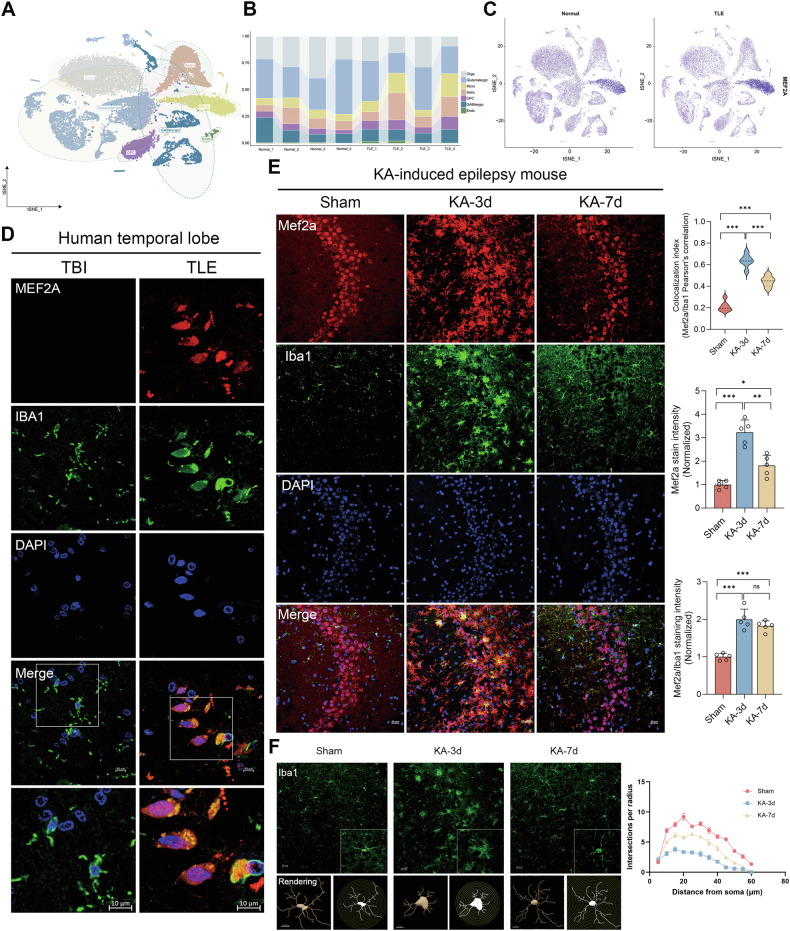
Selective MEF2A upregulation in activated microglia during acute and chronic epilepsy phases. **A** UMAP visualization of single-cell RNA-seq data (GSE190452) annotated using Allen Brain Atlas shows distinct cell clusters (e.g., microglia, neurons) in epilepsy and neurologically normal controls. **B** Each cell’s cluster proportion; **C** FeaturePlot analysis highlights elevated MEF2A expression in microglial clusters of epilepsy. **D** Dual immunofluorescence staining for MEF2A (red) and IBA1 (green) in human epileptogenic foci versus trauma controls (*n* = 6 /group). Scale bar = 10 μm. **E** Dual immunofluorescence staining for Mef2a (red) and Iba1 (green) in mouse hippocampal sections from sham, KA-3d, and KA-7d groups; scale bar = 20 µm (*n* = 5/group), quantifying dual immunofluorescence staining Mef2a expression intensity, Mef2a/Iba1 expression intensity and Pearson’s correlation coefficient for Mef2a/Iba1 co-localization; statistical comparisons between sham and KA-treated groups; **F** 3D reconstruction of microglial morphology using Imaris software based on z-stack imaging (18 slices, 1 µm per slice) and Sholl analysis to quantify ramification; representative images shown (*n* = 5 /group). *, **, *** denote *p* < 0.05, 0.01, 0.001. Data are presented as mean ± SD from each independent experiment. Comparisons between two groups used a two-tailed Student’s *t* test; multi-group comparisons used one-way ANOVA. Scale bars are indicated in representative images. Note: Protein and gene nomenclature follows species-specific guidelines: “MEF2A” refers to human samples (HUGO standards), and “Mef2a” refers to mouse samples (MGI standards).

To elucidate microglia-specific expression patterns of genes associated with epilepsy, we reanalyzed a single-cell RNA sequencing (scRNA-seq) dataset GSE190452, comprising brain tissues from individuals with epilepsy and neurologically normal human controls. Cell cluster annotation was executed using the Azimuth (https://azimuth.hubmapconsortium.org/) as a reference (Fig. 1A). Microglia proportion in patients with epilepsy was significantly higher than that in normal controls (Fig. 1B). Subsequent differential gene expression of patients and controls’ microglia analysis revealed distinct transcriptional profiles. Differentially expressed genes’ (DEGs) temporal dynamics evaluated by integrating murine RNA-seq dataset, GSE99577, including epileptic mice time-series data at 1-, 2-, 4- and 12-days post-induction, revealed that MEF2A exhibited strong co-expression with microglial markers during acute epilepsy phase induction in mice. This association persisted in the chronic phase compared to the controls (Fig. S1A). This indicates that MEF2A may serve as a critical microglial activation regulator in epilepsy. This is further supported by the FeaturePlot visualization of scRNA-seq data, demonstrating increased MEF2A expression within microglial clusters in epileptic samples (Fig. 1C).

Owing to the conserved microglial association of MEF2A across species and experimental paradigms in epilepsy, we characterized its expression patterns. Dual immunofluorescence staining for MEF2A and the microglial marker IBA1 was performed on paraffin-embedded sections from surgically resected temporal lobes of patients with refractory epilepsy (TLE) and intraoperative traumatic brain injury (TBI) controls. This analysis revealed that activated microglia, characterized by an amoeboid morphology, were present in TLE tissues, in contrast to the resting-state microglia observed in the control samples, consistent with previous findings[26]. Notably, robust MEF2A expression was specifically detected in the activated microglia, with significant co-localization of MEF2A and IBA1 (Fig. 1D), whereas resting microglia exhibited minimal MEF2A immunoreactivity. Semi-quantitative immunohistochemical analysis further corroborated the significantly elevated expression of MEF2A in the TLE group compared to the TBI cohort (*p* < 0.001, Fig. S1B). These findings corroborate the results observed in KA-induced mice, where dual immunofluorescence staining for MEF2A and IBA1 demonstrated a significant increase in MEF2A expression (*p* < 0.01 compared to Sham) and enhanced MEF2A/IBA1 co-localization three days post-KA administration (*p* < 0.01 compared to Sham, as shown in Fig. 1E). Although MEF2A expression decreased by day 7, it remained significantly elevated compared to Sham controls (*p* < 0.05; Fig. 1E). In the Sham, KA-3d, and KA-7d groups, no significant changes in MEF2A expression were observed in neurons (*p* > 0.05, Fig. S1C), supporting microglia-specific MEF2A dysregulation. Three-dimensional morphological reconstruction with Sholl analysis revealed dynamic microglial state transitions; activated amoeboid morphology predominated on day-3, whereas ramified morphology resembling the resting state reappeared by day-7 (Fig. 1F). MEF2A upregulation occurred predominantly in amoeboid microglia during the acute phase (Fig. 1D–F). These findings align with the analysis of single-cell sequencing data, further elucidating the strong association between activated microglia and MEF2A in the context of refractory epilepsy.

### ATP-Ca²^+^ axis mediates neuronal hyperactivity-induced microglial MEF2A activation

**Fig. 2:**
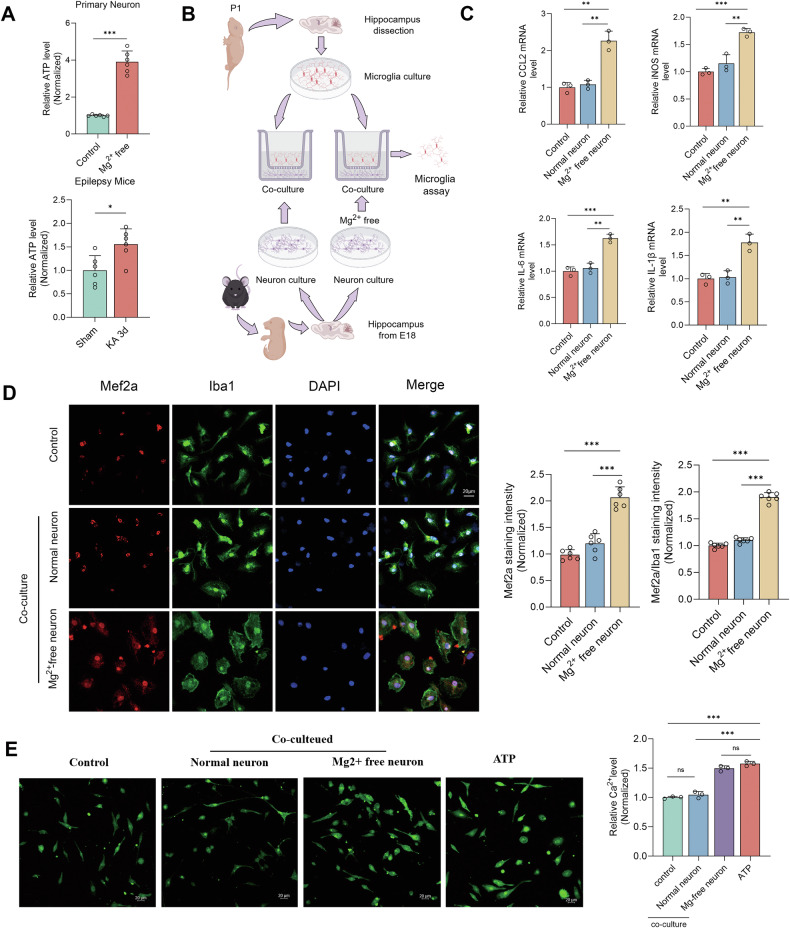
Role of ATP in microglial activation and calcium signaling in epilepsy models. **A** ATP levels in in vivo (sham-operated mice vs KA-induced epilepsy mice) and in vitro epilepsy models (normal vs Mg^2^⁺-free medium-exposed neurons). ATP was quantified using a luciferin-luciferase-based assay (*n* = 6; analyzed by Student’s *t* test); **B** Experimental design schematic: Primary hippocampal neurons (E18 mice) were exposed to Mg^2^⁺-free medium to induce epilepsy-like activity. Primary microglia (P0) were co-cultured with either normal or epilepsy-inducing neurons to establish co-culture systems; **C** Relative mRNA expression of pro-inflammatory cytokines (CCL2, iNOS, IL-6, IL-1β) in microglia from control groups, normal neuron co-cultures, and epilepsy-induced neuron co-cultures (*n* = 3; analyzed by one-way ANOVA); **D** Representative immunofluorescence images of Mef2a (red) and Iba1 (green) co-localization in microglia. Quantifying Mef2a fluorescence intensity and Mef2a/Iba1 co-localization (mean fluorescence intensity, *n* = 6; *p < 0.001 for all comparisons), scale bar = 20 µm; **E** Fluorescent images of intracellular Ca^2^⁺ levels (green, Fluo4 dye) in microglia from control groups, normal neuron co-cultures, epilepsy-induced neuron co-cultures, and ATP-treated microglia, scale bar = 20 µm; Quantifying relative Ca^2^⁺ fluorescence intensity (*n* = 3; analyzed by one-way ANOVA). *, **, *** denote significance levels (*p* < 0.05, 0.01, 0.001) compared to the control group or specified comparison groups (e.g., normal vs. epilepsy-induced neurons). Comparisons between two groups used a two-tailed Student’s *t* test; multi-group comparisons used a one-way ANOVA with a Bonferroni post hoc test for pairwise comparisons. Data are presented as mean ± SD from three independent experiments. Scale bars are indicated in representative images.

Given the spatiotemporal correlation between MEF2A dynamics and seizure-associated microglial activation, we investigated the hypothesis that epileptiform activity-induced ATP release activates microglial purinergic receptors, leading to calcium-dependent MEF2A upregulation by quantifying ATP dynamics using luciferase-based bioluminescence assays in in vivo and in vitro epilepsy models. In vivo, hippocampal ATP concentrations were elevated on day-3 post-KA administration compared to Sham controls; In vitro, Mg^2+^-free medium-induced epileptiform activity in primary neurons resulted in increased ATP release compared to that in untreated neurons (Fig. 2A).

Using a Transwell co-culture system to integrate primary neurons and microglia for investigating the crosstalk between them during epileptiform activity (Fig. 2B). Mature neurons were identified through immunofluorescence staining using MAP2/Neun markers, as illustrated in Fig. S2A. Neuronal hyperactivity was induced by subjecting the neurons to a Mg^2+^-free medium for a duration of 3 h. Quantitative real-time PCR (qRT-PCR) analysis revealed a significant upregulation of microglial activation markers, including CCL2, inducible nitric oxide synthase (iNOS), IL-6, and IL-1β, in co-cultures where neurons were treated in Mg^2+^-free medium, as compared to co-cultures with neurons in normal medium (*p* < 0.01 for all markers; refer to Fig. 2C). Immunofluorescence analysis showed increased MEF2A protein expression (*p* < 0.01) and extensive co-localization of MEF2A -positive cells with Iba-1 (Fig. 2D). Additionally, MEF2A /Iba-1 staining intensity in microglia co-cultured with neurons exposed to Mg^2+^-free environment was significantly enhanced, further associating MEF2A expression with neuronal hyperactivity-induced microglial activation (Fig. 2D). This suggest that epileptic neuronal hyperexcitability triggers microglial activation, which is closely associated with microglial MEF2A expression. To validate whether calcium signaling is activated by extracellular ATP released from epileptic neurons, we quantified intracellular calcium dynamics. Microglia exposed to ATP released from epileptiform neurons or directly applied ATP (800 μM; simulating extracellular conditions following epileptic activity) exhibited similar elevated calcium levels (*p* > 0.05; Fig. 2E), with both conditions exhibiting significantly higher calcium levels than the control group (Fig. 2E).

**Fig. 3:**
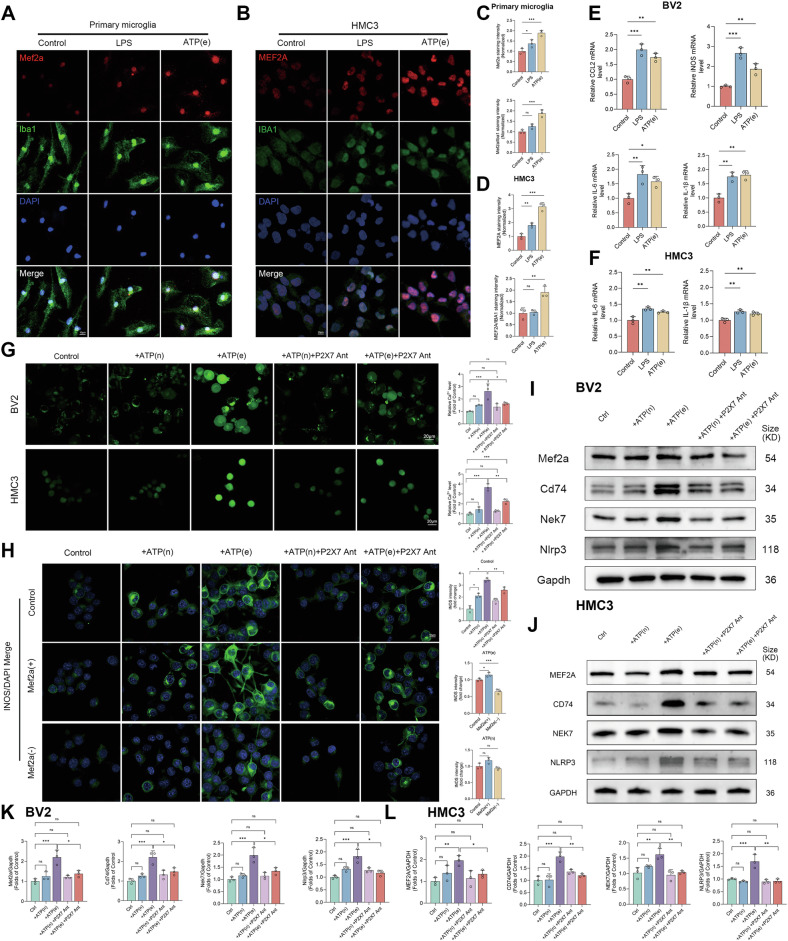
ATP activated the MEF2A-NEK7/CD74 axis through P2X7 receptor-mediated Ca2+ signaling in BV2 and HMC3 cell lines. **A**, **B** Shown are representative immunofluorescence images depicting the co-localization of MEF2A (red) and IBA1 (green) in control, LPS, and extracellular ATP (ATP(e)) conditions, which simulate epileptic activity, within primary microglia and HMC3 cell lines. scale bar = 10 µm. **C**, **D** The quantification of MEF2A fluorescence intensity and the extent of MEF2A/IBA1 co-localization in primary microglia and the HMC3 cell line are reported as mean fluorescence intensity (*n* = 3). All comparisons demonstrated statistically significant differences (**p < 0.01). **E** Relative mRNA levels of CCL2, iNOS, IL-6, and IL-1β in control, LPS-treated, and epilepsy-ATP-exposed BV2 cell line (analyzed by one-way ANOVA, *n* = 3). **F** Relative mRNA levels of IL-6, and IL-1β in control, LPS-treated, and epilepsy-ATP-exposed HMC3 cell line (analyzed by one-way ANOVA, *n* = 3). **G** Fluorescent images of intracellular Ca²⁺ levels (Fluo4 dye) in BV2 and HMC3 cells under control conditions, normal extracellular ATP (ATP(n)), epilepsy-mimicking ATP (ATP(e)), ATP(n) pretreated with P2X7 antagonist, and ATP(e) pretreated with P2X7 antagonist, the fluorescence intensity in each group was quantified. (*n* = 3, scale bar = 20 µm). **H** Immunofluorescence images depicting inducible nitric oxide synthase (iNOS) in green, merged with DAPI staining, are presented for control, MEF2A-overexpressing (MEF2A(+)), and MEF2A-deficient (MEF2A (–)) BV2 cells, all subjected to identical experimental conditions as described in (**G**) (*n* = 3, scale bar = 10 µm). **I**, **J** Western blot analysis was conducted to assess the expression levels of MEF2A, CD74, NEK7, NLRP3, and GAPDH (as a loading control) in BV2 and HMC3 cells, under the conditions outlined in (**G**). **K**, **L** Represent semi-quantitative analyses and statistical charts corresponding to the Western blot images presented in (**I**, **J**). Data are presented as mean ± SD from three independent experiments, with statistical comparisons performed using two-tailed Student’s *t* test or one-way ANOVA (*p < 0.05, **p < 0.01, ***p < 0.001 vs. control or specified comparison groups). Note: Protein and gene nomenclature follows species-specific guidelines: “MEF2A” refers to human samples (HUGO standards), and “Mef2a” refers to mouse samples (MGI standards).

Subsequently, we investigated the distinct mechanisms through which inflammatory and ATP-driven pathways contribute to the upregulation of MEF2A. To thoroughly investigate the ATP-mediated regulation of MEF2A across species, our analysis was expanded to include murine primary microglia and human (HMC3) cell lines [36, 37]. This was achieved by exposing primary microglia and HMC3 cells to low-dose lipopolysaccharide (LPS, 100 ng/mL) or extracellular ATP (800 μM; to simulate conditions following epileptic activity) for a duration of 24 h. Both treatments resulted in similar amoeboid morphological changes in primary microglia, as demonstrated by IBA1 staining (Fig. 3A, C). Remarkably, ATP-treated microglia exhibited a significant increase in MEF2A expression in primary microglia and HMC3 cells compared to the control or LPS-treated groups (*p* < 0.01; Fig. 3B, D). This finding suggests that MEF2A expression is more closely associated with ATP signaling pathways rather than with LPS. To further elucidate the differential activation patterns elicited by LPS versus ATP, we quantified the expression of key inflammatory mediators in BV2 and HMC3 cells using qPCR. Specifically, BV2 cells were analyzed for the expression of CCL2, iNOS, IL-6, and IL-1β, whereas HMC3 cells were examined for IL-6 and IL-1β expression. Both stimuli significantly elevated the levels of these inflammatory mediators compared to controls, and distinct cytokine induction profiles were observed between the treatments (Fig. 3E, F). This divergence in cytokine expression underscores the distinct pathways activated by ATP and LPS in BV2 and HMC3 cells, with ATP signaling preferentially enhancing MEF2A expression.

Since experiments conducted on primary microglia demonstrated that the calcium response of activated microglia closely resembled that induced by direct ATP stimulation (Fig. 2E). To further validate the direct relationship between ATP and calcium ions (Ca^2+^) influx, we examined the effects of P2X7 receptor antagonists on intracellular Ca^2+^ levels in ATP-treated BV2 and HMC3 cells. The P2X7 receptor antagonists significantly diminished the ATP-induced increase in intracellular calcium levels (Fig. 3G). Additionally, we explored whether ATP-driven Ca^2+^ signaling activated microglia via the MEF2A pathway. In BV2 cells with MEF2A knockdown (MEF2A (–)), ATP stimulation coupled with iNOS expression was significantly reduced compared to controls (*p* < 0.01; Figs. 3H and S2B illustrates the channel split diagram). Conversely, in MEF2A-overexpressing cells (MEF2A ( + )), ATP treatment resulted in a higher increase in iNOS expression than in controls (*p* < 0.05; Figs. 3H and S2B). Notably, the P2X7 receptor antagonist A-438079 inhibited ATP-induced iNOS upregulation across all groups (*p* < 0.01; Figs. 3H and S2B).

These studies demonstrate that ATP induces microglial activation through a pathway dependent on the P2X7 receptor, Ca^2⁺^, and MEF2A. Furthermore, the findings reveal that neuronal hyperactivity elevates extracellular ATP levels, which subsequently promotes the upregulation of MEF2A in microglia through calcium-dependent purinergic signaling, independent of the conventional LPS-activated inflammatory pathways.

### MEF2A regulates microglial activation via CD74 and drives NEK7-dependent upregulation of NLRP3

This in-depth investigation of the molecular mechanisms by which MEF2A modulates microglial activation through ATP signaling. Initially, we validated the hypothesis that ATP-induced Ca^2^⁺ influx directly influences MEF2A regulation and its downstream molecule expression. Predictions from transcription factor databases (Fig. S3) suggested that MEF2A directly regulates kinase NEK7 transcription, whereas microglial-activated marker CD74, with NEK7, plays a pivotal role in NLRP3 synthesis. To elucidate the relationship between ATP-mediated Ca²⁺ influx and MEF2A expression, and to assess whether MEF2A orchestrates changes in CD74, NEK7, and NLRP3, we inhibited the ATP/P2X7 signaling pathway using the P2X7 receptor antagonist A-438079 at a concentration of 20 μM. The experimental design groups were: (1) untreated normal controls; (2) ATPn at 100 μM; (3) epileptic-like ATPe at 800 μM; (4) ATPn combined with A-438079; and (5) ATPe combined with A-438079. Western blot (WB) analyses were conducted after a 24-h treatment period.

In murine (BV2) and human (HMC3) cell lines, the study demonstrated that treatment with ATPn showed non-significant alteration in MEF2A protein levels compared to the control group (*p* > 0.05, Fig. 3I–L). Conversely, ATPe treatment significantly upregulated MEF2A (*p* < 0.01 compared to ATPn, Fig. 3I–L). Co-incubation with A-438079 completely negated the ATPe-induced increase in MEF2A, restoring levels to those observed in controls (*p* > 0.05 compared to controls, Fig. 3I–L), and significantly inhibited the ATPe-induced increase in intracellular Ca^2^⁺ levels (*p* < 0.01 compared to ATPe alone, Fig. 3G). These results substantiate that MEF2A expression is contingent on Ca^2^⁺ influx mediated by ATP/P2X7 receptor.

Moreover, we revealed that MEF2A protein dynamics positively correlated with target gene expression. Specifically, ATPe significantly increased the protein levels of CD74, NEK7, and NLRP3 compared to ATPn (*p* < 0.01 for all comparisons) in BV2 and HMC3 cell lines. This effect was reversed by preincubation with A-438079, resulting in no significant difference from ATPn (*p* > 0.05) (Fig. 3I–L). Collectively, these findings indicate that MEF2A facilitates microglial activation through the coordinated upregulation of CD74, NEK7, and NLRP3, a process that is critically dependent on ATP/P2X7-Ca²⁺ signaling.

**Fig. 4:**
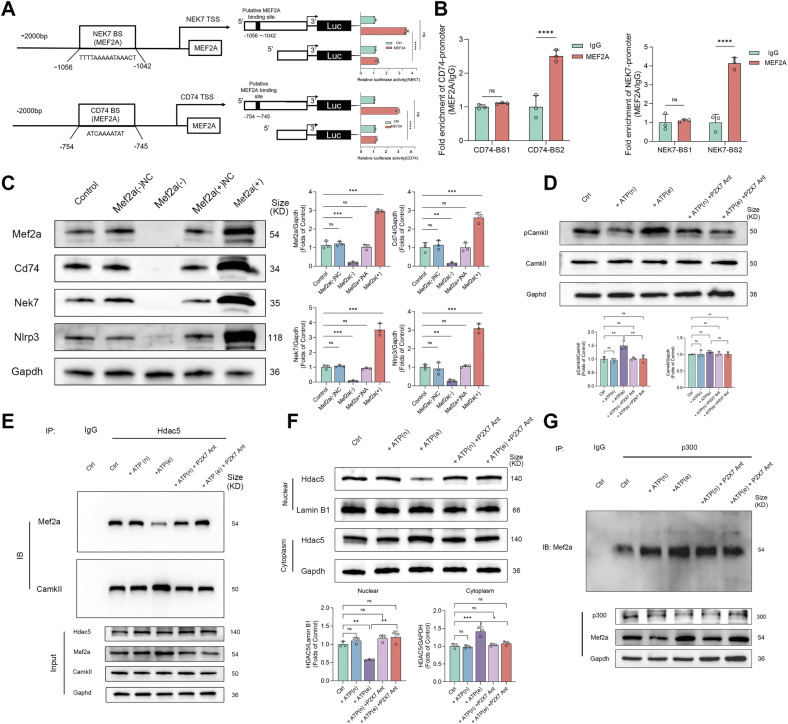
ATP stimulates the HDAC5/CaMK2-MEF2A pathway via the P2X7 receptor, enhancing MEF2A’s binding to NEK7 and CD74 promoters and increasing their expression, which triggers neuroinflammation. This process can be inhibited by P2X7 antagonists. **A** Dual-luciferase reporter assays demonstrating the binding of MEF2A to the promoter regions of NEK7 and CD74 in HEK293T cells, *n* = 3. **B** Chromatin immunoprecipitation (ChIP) analysis confirming MEF2A occupancy at the promoter regions of NEK7 and CD74 in HEK293T cells (fold enrichment relative to input%), *n* = 3. **C** Western blot analysis of Mef2a, Cd74, Nek7, Nlrp3, and Gapdh (loading control) in control BV2 cells, Mef2a(-)NC (Mef2a knockdown with non-targeting control), Mef2a(-) (Mef2a knockdown), Mef2a(+)-NC (Mef2a overexpression with non-targeting control), and Mef2a(+) (Mef2a overexpression) groups; quantitative protein levels are shown; *n* = 3. **D** Western blot quantification of phosphorylated (pCamkII) and total CamkII levels under control, normal extracellular ATP (ATP(n)), epilepsy-mimicking ATP (ATP(e)), ATP(n) pretreated with P2X7 antagonist, and ATP(e) pretreated with P2X7 antagonist conditions, *n* = 3. **E** Co-IP assays measuring interactions between Hdac5 and Mef2a/CamkII in BV2 cells in the same experimental groups, *n* = 3. **F** Western blot analysis of nuclear and cytoplasmic Hdac5 protein expression in the groups described in (**D**), *n* = 3. **G** Co-IP assays assessing p300-MEF2A interactions under the same experimental conditions as (**D**), *n* = 3. Data represent mean ± SD from three independent experiments; statistical comparisons were performed using two-tailed Student’s t-test or one-way ANOVA (*p < 0.05, **p < 0.01, ***p < 0.001, ****p < 0.0001 vs. control or indicated comparison groups).

To determine whether MEF2A directly regulates the transcription of NEK7 and CD74 by binding to their promoter regions, we conducted luciferase reporter assays. In HEK293T cells, co-transfection with plasmids containing NEK7 or CD74 wild-type promoters, along with MEF2A overexpression vectors, significantly increased luciferase activity (NEK7: *p* < 0.01; CD74: *p* < 0.001 compared to empty vector controls). Conversely, promoter constructs lacking MEF2A -binding sites exhibited no significant changes in activity (*p* > 0.05) (Fig. 4A). Additionally, chromatin immunoprecipitation (ChIP) assays revealed that MEF2A antibodies significantly enriched DNA fragments from the NEK7 (*p* < 0.01) and CD74 (*p* < 0.001) promoter regions in HEK293T (Fig. 4B), whereas rabbit IgG controls did not display any detectable binding signals. These results confirm that MEF2A directly binds to the promoters of NEK7 and CD74, thereby activating their transcription, with the binding sites corroborated by reporter and ChIP assays.

To further elucidate MEF2A transcriptional regulatory function on CD74 and NEK7 in microglia, we generated stable MEF2A(-) and MEF2A(+) cell lines via lentiviral transduction and puromycin selection in BV2 cells. Specifically, stable knockdown was achieved using a MEF2A-specific shRNA (MEF2A (–)), with a non-targeting shRNA serving as the control (MEF2A(-)NC), and overexpression via lentiviral vector encoding MEF2A (MEF2A(+)), along with an empty vector control (MEF2A( + )NC). WB analysis revealed significantly reduced MEF2A protein levels in MEF2A (–) cells compared to MEF2A(-)NC (*p* < 0.001), whereas MEF2A(+) cells exhibited marked MEF2A increase compared to MEF2A( + )NC(*p* < 0.001). Subsequent target protein quantification indicated that MEF2A(-) suppressed CD74, NEK7, and NLRP3 expressions. Specifically, MEF2A (–) cells showed significantly reduced CD74 (*p* < 0.01), NEK7 (*p* < 0.001), and NLRP3 (*p* < 0.05) levels compared with MEF2A(-)NC (Fig. 4C). Conversely, MEF2A(+) cells showed significantly elevated CD74 (*p* < 0.01), NEK7 (*p* < 0.001), and NLRP3 (*p* < 0.05) levels compared with MEF2A( + )NC (Fig. 4C).

These findings are consistent with those of the dual-luciferase reporter (Fig. 4A) and ChIP assays (Fig. 4B), confirming the direct transcriptional activation of CD74 and NEK7 by MEF2A. Notably, the observed NEK7 and NLRP3 expression correlation (*p* < 0.05) aligns with NEK7’s established function as a kinase facilitating NLRP3 maturation, as previously documented [38]. Collectively, these data substantiate MEF2A’s dual role in directly promoting CD74 and NEK7 transcription while indirectly modulating NLRP3 expression through NEK7-dependent pathways.

### ATP-mediated HDAC5-dependent MEF2A acetylation is critical for its transcriptional activation

To elucidate the mechanism by which ATP modulates MEF2A expression and activity, we initially focused on the established HDAC5 role in transcriptional MEF2A regulation. HDAC5 is associated with the MEF2A N-terminal region, thereby repressing its transcriptional activity [39]. However, stress signals, including CAMKII-mediated phosphorylation of serine residues on HDAC5, facilitate HDAC5 dissociation from MEF2A [40]. This enables MEF2A interaction with histone acetyltransferases (HATs), thereby promoting downstream gene transcription [41]. For our study, experimental ATP concentrations elicited Ca2⁺ influx in BV2 cells; thus, we examined Ca2⁺-dependent CAMKII phosphorylation and demonstrated that ATP significantly elevated phosphorylated CAMKII levels (Fig. 4D), an effect that was entirely inhibited by a P2X7 receptor antagonist (Fig. 4D). These results suggest that ATP-induced Ca2⁺ influx directly facilitates CAMKⅡ phosphorylation by activating P2X7 receptor.

To further explore activated CAMKⅡ impact on HDAC5, co-immunoprecipitation (Co-IP) experiments demonstrated that ATP treatment significantly enhanced CAMKⅡ and HDAC5 interaction (Fig. 4E). This effect was reversed by treatment with P2X7 receptor antagonist (Fig. 4E). Additionally, subcellular localization analysis showed constant total HDAC5 protein levels (Fig. 4E), whereas ATP treatment showed markedly decreased nuclear HDAC5 and corresponding cytoplasmic HDAC5 increase (Fig. 4F). This nuclear-to-cytoplasmic translocation was completely reversed following treatment with P2X7 receptor antagonist (Fig. 4F). This suggest that CAMKⅡ binding facilitates cytoplasmic HDAC5 retention. Notably, Co-IP experiments demonstrated markedly reduced interaction between HDAC5 and MEF2A with ATP treatment (Fig. 4E), an effect reversible by administering a P2X7 antagonist. This finding implied that HDAC5 translocation to the cytoplasm alleviated its inhibitory influence on MEF2A, thereby facilitating MEF2A's role in transcriptional activation. Similarly, ATP treatment significantly enhanced the interaction between MEF2A and the HAT p300 (Fig. 4G). This strongly suggests that HDAC5 dissociation alleviates transcriptional repression, permitting MEF2A acetylation and subsequent transcriptional activation.

Collectively, we show that ATP activates Ca^2^⁺ influx through P2X7 receptors, leading to CAMKⅡ activation and its interaction with HDAC5. This interaction facilitates HDAC5 movement from the nucleus to the cytoplasm, a process reversible by administering a P2X7 receptor antagonist. This relocation reduces HDAC5’s association with MEF2A, enhancing MEF2A's interaction with p300 and its transcriptional activity. Thus, ATP-induced Ca^2^⁺ signaling via P2X7 receptors reorganizes HDAC5, relieving MEF2A repression and enhancing its transcriptional activity.

### Parecoxib interacts with MEF2A to suppress its transcriptional activity

**Fig. 5:**
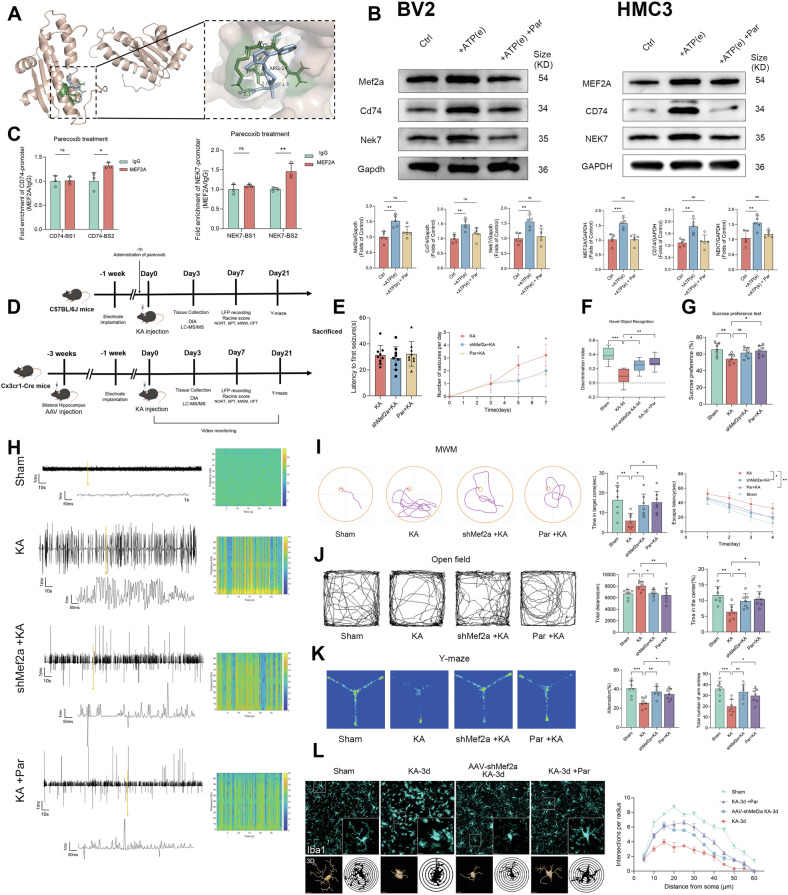
Targeted MEF2A treatment alleviates epileptic seizure activity and hippocampal microglial responses. **A** Molecular docking analysis of parecoxib and MEF2A. **B** Western blot analysis of MEF2A, CD74, NEK7, and GAPDH protein expression in BV2 cells and HMC3 cells under control, ATP(e) treatment, and ATP(e)+parecoxib treatment conditions. Quantified protein expression data analyzed by one-way ANOVA; *n* = 5 per group. **C** ChIP assay showing MEF2A binding to NEK7 and CD74 promoters in HEK293T cells after parecoxib treatment; analyzed by Student’s *t* test; *n* = 3 technical replicates. **D** Experimental design for KA-induced epilepsy model with AAV-shMef2a and parecoxib treatments. **E** Seizure latency and daily seizure frequency in KA, AAV- shMef2a+KA, and parecoxib+KA groups; analyzed by two-way ANOVA; *n* = 8 per group. **F** Novel object recognition (NORT) test results in Sham, KA, AAV- shMef2a+KA, and parecoxib+KA groups; analyzed by one-way ANOVA; *n* = 8 per group. **G** Sucrose preference test results in Sham, KA, AAV- shMef2a+KA, and parecoxib+KA groups; analyzed by one-way ANOVA; *n* = 7 per group. **H** Representative hippocampal LFP recordings and spectral analysis in Sham, KA, AAV- shMef2a+KA, and parecoxib+KA groups; *n* = 6 per group. **I** The swimming trajectory maps and performance metrics (escape latency and platform crossings) in the Morris Water Maze (MWM) were assessed among the Sham, KA, AAV- shMef2a+KA, and parecoxib+KA groups. The data were analyzed using a one-way ANOVA, with a sample size of *n* = 6 for each group. **J** Representative locomotor trajectory plots, along with quantification of the total distance traveled and the time spent in the center area during the open field test, were analyzed among the Sham, KA, AAV- shMef2a+KA, and parecoxib+KA groups. The data were evaluated using a one-way ANOVA, with a sample size of *n* = 7 per group. **K** Representative movement heat maps and the quantification of alternation rates and total arm entries in the Y-maze test were assessed among the Sham, KA, AAV- shMef2a +KA, and parecoxib+KA groups. The data were analyzed using a one-way ANOVA, with a sample size of *n* = 7 per group. **L** Representative Iba1 immunostaining was conducted to examine the morphology of hippocampal microglia, along with a 3D reconstruction of microglia in the Sham, KA, AAV- shMef2a+KA, and parecoxib+KA groups. The scale bar represents 20 µm. Sholl analysis was utilized to assess microglial branching complexity, and the data were analyzed using one-way ANOVA, with a sample size of *n* = 6 cells per group. Data are presented as mean ± SD from six independent experiments unless stated otherwise. Statistical comparisons were performed using two-tailed Student’s *t* test or one-way/two-way ANOVA; Significance levels: *p < 0.05, **p < 0.01, ***p < 0.001 vs. control or indicated comparison groups.

The N-terminal α1 helix, comprising residues ARG17, THR20, PHE21, LYS23, and ARG24, as well as the MADS-box domain of MEF2A, is an essential structural element for DNA binding. α1 helix undergoes significant conformational changes during DNA interaction, particularly at residues LYS23 and ARG24, contributing to the destabilization of MEF2A -DNA interface [42]. Thus, these residues are viable targets for inhibitor development. Considering the lack of human safety validation of previously reported MEF2A inhibitors, including inhibitor A and BML-210, we screened structurally similar compounds. As molecular docking simulations revealed, parecoxib specifically interacts with LYS23, ARG24, and THR20 residues of MEF2A. Based on binding energy analyses, these interactions exhibited a high affinity (Fig. 5A), suggesting that parecoxib may inhibit MEF2A function by obstructing its DNA-binding capability.

To validate the inhibitory parecoxib effects on MEF2A -mediated microglial activation and transcriptional activity, cytotoxicity was evaluated. MTT assays showed parecoxib concentrations of ≤10 μM showed no significant toxicity in BV2, HMC3 and HEK293T cells, with cell viability exceeding 95% (*p* > 0.05, Fig. S4A). Subsequent experiments demonstrated that 80 μM parecoxib significantly attenuated ATP (800 μM)-induced expression of iNOS in BV2 cells (*p* < 0.05; Fig. S4B, C). Based on these findings, 80 μM parecoxib was selected for further studies. Co-treatment with ATP (800 μM, 24-h stimulation) revealed that parecoxib inhibited ATP-induced MEF2A protein upregulation (*p* < 0.01) and its downstream targets NEK7 and CD74 (*p* < 0.05) in BV2 and HMC3 cells, as determined by WB analysis (Fig. 5B). ChIP-qPCR analysis further demonstrated that parecoxib reduced MEF2A DNA occupancy at the NEK7 promoter (from 3.9-fold to 1.5-fold, *p* < 0.01) and the CD74 promoter (from 2.2-fold to 1.3-fold, *p* < 0.01) in HEK293T cells (Fig. 5C).

These findings suggested that parecoxib inhibits transcriptional activity by specifically targeting its DNA-binding interface, thereby highlighting its potential as an MEF2A inhibitor. To assess whether parecoxib’s effects stem from MEF2A inhibition rather than its nonsteroidal anti-inflammatory drug (NSAID) properties, we tested celecoxib, which does not bind MEF2A. In BV2 and HMC3 cells, celecoxib did not significantly affect ATP-induced increases in MEF2A, NEK7, or CD74 (Fig. S5A), nor did it alter seizure frequency in KA-induced epileptic mice (Fig. S5B). This indicates that parecoxib’s effectiveness is primarily due to MEF2A inhibition, not COX enzyme inhibition. Furthermore, the COX enzyme inhibitory properties of parecoxib may augment its therapeutic potential for treating neuroinflammatory diseases. To assess in vivo therapeutic efficacy, we implemented two interventions in the KA-induced epilepsy mouse (KA-3d) model. The experimental groups comprised Sham-operated controls, KA-3d, and KA mice treated with either intracerebroventricular (i.c.v.) parecoxib injection or AAV-shMEF2A-mediated microglial-specific MEF2A knockdown in the hippocampal CA3 region. The intraventricular injection concentration was determined by a pre-experiment (Fig. S5C). We subsequently evaluated the interventions’ impact on chronic-phase epileptic seizure frequency, severity of electroencephalographic abnormalities during seizure, cognitive and memory functions in epileptic mice, and microglia neuroinflammation in epileptic mice to determine whether targeting MEF2A could exert therapeutic effects in epileptic mice.

### Targeted MEF2A treatment alleviates epileptic seizure activity and hippocampal microglial responses

To evaluate whether targeting microglial MEF2A mitigated KA-induced temporal lobe epilepsy (TLE) and associated cognitive deficits in C57BL/6 mice, we conducted experiments outlined in Fig. 5D. As Methods section shows, mice were allocated into (1) Sham-operated group (received intramygdaloid saline injection, with equivalent volume to the KA group, via stereotaxic surgery); (2) KA-induced TLE C57BL/6 mice group which received stereotaxic KA injection; (3) KA+shMEF2A 3w Cx3cr1-Cre mice group first subjected to bilateral intrahippocampal AAV-shMEF2A infusion (targeting CA3 region) to achieve microglial-specific MEF2A knockdown, followed by KA injection into the amygdala 4 weeks later; and (4) parecoxib administration (KA+Par) C57BL/6 mice group, which received parecoxib i.c.v. injection 1 h before the stereotaxic amygdala KA injection.

Although no significant difference occurred in the latency to first seizure onset across all experimental groups (Fig. 5E), KA+shMEF2A and KA+Par treatment groups exhibited significantly reduced daily seizure frequency during the chronic phase compared to the KA group (Fig. 5E; *p* < 0.05, both). Importantly, the two treatment groups differed non-significantly (Fig. 5E; *p* > 0.05), suggesting comparable efficacy in reducing seizure recurrence. To assess electrographic alterations, we recorded hippocampal local field potential (LFP) 7 days after KA injection. Spectral analysis of LFP power (0–100 Hz frequency range) showed pronounced high-frequency oscillations (HFOs) in KA mice, particularly within the γ-band (30–100 Hz); indicative of epileptiform activity (Fig. 5H). Both treatment groups showed significant reductions in ictal electrographic spikes and marked HFO power suppression. Specifically, γ-band power diminished in KA+shMEF2A and KA+Par mice compared to KA mice (Fig. 5H). These findings align with existing evidence on therapies targeting MEF2A, including shMEF2A, which significantly reduced epileptic seizure frequency and severity in mice, as behavioral and electrophysiological measures showed.

Given that targeting MEF2A treatment has the potential to mitigate the severity of epileptic seizures in KA-induced epilepsy in mice, we conducted a further investigation into the effects of these treatments on epilepsy-related comorbidities, including cognitive impairment, depression, spatial learning and memory disorders, anxiety, and working memory deficits. At 7 days following KA injection, significant behavioral deficits were observed. The novel object recognition test (NORT) demonstrated an impaired discrimination index (*p* < 0.001 compared to Sham, Fig. 5F), whereas the Morris water maze (MWM) revealed prolonged escape latency (*p* < 0.01) and reduced time in the target zone (*p* < 0.05) (Fig. 5I). The open field test indicated decreased time spent at the center and reduced total distance traveled (both *p* < 0.05, Fig. 5J), and the sucrose preference test suggested depressive-like behavior (*p* < 0.01, Fig. 5G). Treatments with shMEF2A and parecoxib mitigated these deficits, as evidenced by the significantly improved NORT discrimination indices, MWM performance parameters, and open field measures compared to the KA group (*p* < 0.05 for all), although neither treatment fully restored function to Sham levels (*p* < 0.05) (refer to Fig. 5F–J). Notably, parecoxib, but not shMEF2A, alleviated sucrose preference deficits (*p* < 0.05 vs. KA; shMEF2A *p* > 0.05, Fig. 5G). At the 21-day assessment, Y-maze testing showed that KA caused working memory and exploratory drive impairments, with lower alternation rates (*p* < 0.05) and fewer total number of arm entries (*p* < 0.01) compared to the Sham group. Both treatments successfully reversed these deficits, improving alternation rates and arm entries compared to the KA group (*p* < 0.05), with no significant differences between the treatments (all *p* > 0.05) (Fig. 5K).

To evaluate microglial activation during acute seizures, we used IBA1 immunostaining and Sholl analysis to examine microglial morphology changes (Fig. 5L). In the Sham group, microglia had a typical ramified shape with small somas and many branches. After KA-induced status epilepticus (SE), untreated mice showed significant microglial activation, with larger somas and fewer branches. However, treated groups (KA+shMEF2A and KA+Par) showed reduced activation compared to untreated SE mice (*p* < 0.01). Sholl analysis indicated that treated groups maintained better branching complexity up to 60 µm from the soma, with the KA+shMEF2A group having more branch intersections than untreated mice (*p* < 0.05). Treated mice had smaller somas than untreated ones (*p* < 0.01), but still larger than control mice (*p* < 0.001) (Fig. 5L). Furthermore, microglial density in the CA3 region was significantly higher in all SE groups than in the control group (*p* < 0.001). Treatment reduced microgliosis compared to untreated mice (*p* < 0.05), but cell counts were still notably higher than in the control group (*p* < 0.01) (see Figs. 5L and S5D). The results of detecting the IL-1β mRNA level in the CA3 region of the mouse hippocampus showed that both treatments could significantly reduce the expression of IL-1β in the hippocampus (Fig. S5E). Collectively, these indicate that both therapeutic interventions mitigate excessive microglial activation induced by SE, although functional microglial morphology recovery and density remain incomplete. This is consistent with the persistent hippocampal synaptic dysfunction observed in behavioral assays.

KA+shMEF2A and KA+Par both effectively reduced KA-induced seizures, epilepsy-related issues, and microglial hyperactivation, showing similar benefits like fewer seizures, improved memory, and normalized microglial shape. However, neither fully resolved hippocampal issues, as microgliosis and some cognitive deficits remained. These findings highlight MEF2A’s crucial role in controlling microglial-driven epilepsy and cognitive problems.

### Microglial MEF2A downregulation reverses inhibitory synaptic protein loss

**Fig. 6:**
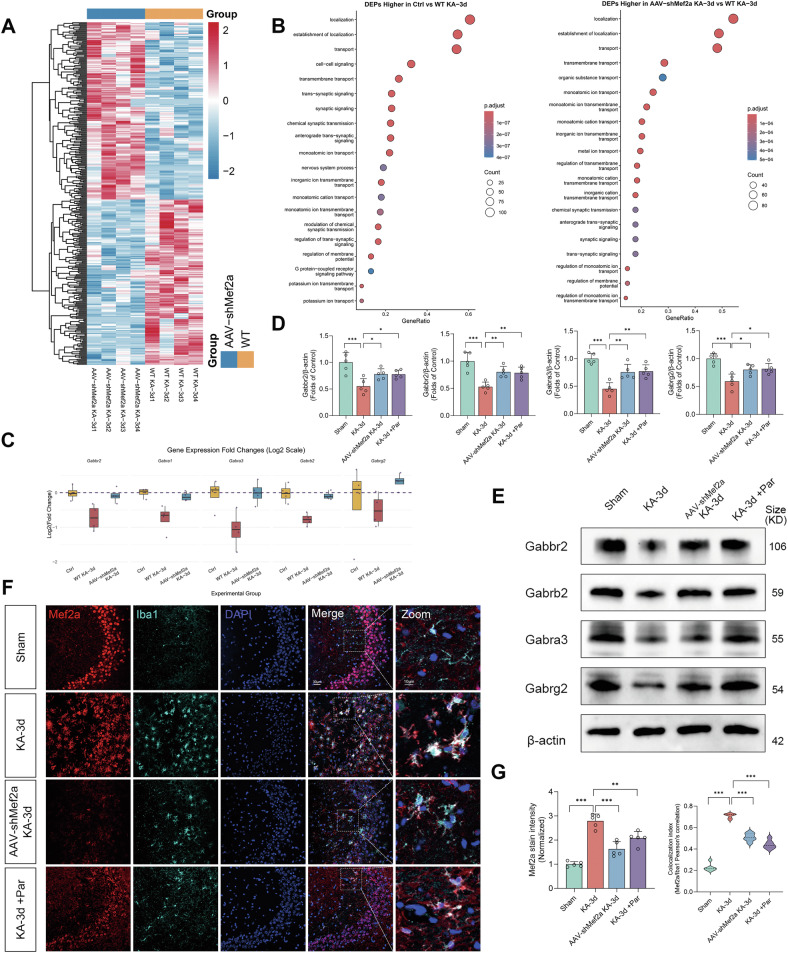
Target MEF2A treatment reverses inhibitory synaptic protein loss. **A** Heatmap of differentially expressed proteins between WT KA-3d and AAV- shMef2a KA-3d groups; **B** GO clustering analysis of differentially expressed proteins between WT KA-3d vs AAV- shMef2a KA-3d and Ctrl vs WT KA-3d groups; **C** Boxplot analysis of Gabbr1, Gabra1, Gabra3, Gabrb2, and Gabrg2 protein expression levels in Ctrl, WT KA-3d, and AAV- shMef2a KA-3d groups; analyzed by one-way ANOVA; **D,** **E** Western blot analysis of Gabbr2, Gabrb2, Gabra3, and Gabrg2 protein expression in hippocampal tissues from sham, WT KA-3d, AAV- shMef2a KA-3d, and par+KA-3d groups; *n* = 5 per group; **F** Dual immunofluorescence staining for Mef2a (red) and Iba1 (cyan) in hippocampal sections from sham, WT KA-3d, AAV- shMef2a KA-3d, and par+KA-3d groups; scale bar = 20 µm; *n* = 5 per group; **G** Quantification of Mef2a expression intensity and Pearson’s correlation coefficient for Mef2a/Iba1 co-localization in **F**; analyzed by one-way ANOVA; statistical comparisons between sham and treatment groups. Data are presented as mean ± SD from three independent experiments unless stated otherwise. Statistical comparisons were performed using a two-tailed Student’s *t* test or one-way ANOVA. Significance levels: *p < 0.05, **p < 0.01, ***p < 0.001 vs. control or indicated comparison groups.

To further investigate the molecular mechanisms by which microglial MEF2A regulates seizure activity and cognitive deficits, we conducted proteomic analysis on Sham, KA, and KA+shMEF2A mice hippocampal tissues during the acute phase (3 days post-KA injection). We identified 166 downregulated and 178 upregulated proteins in the KA+shMEF2A group compared to those in the KA group (Fig. 6A shows distinct clustering patterns in a heatmap). Comparisons between Sham and KA groups revealed 213 downregulated and 782 upregulated proteins, whereas comparisons between Sham and KA+shMEF2A groups revealed 407 downregulated and 89 upregulated proteins, respectively (Fig. S6A–F). Gene Ontology enrichment analysis of differentially expressed proteins in both KA vs KA+shMEF2A and Sham vs KA comparisons revealed significantly enriched synaptic-related pathways (Fig. 6B). Further analysis of inhibitory synaptic receptor proteins using fold-change comparisons revealed significantly downregulated Gabbr2, Gabra1, Gabra3, Gabrb2, and Gabrg2 were in KA-3d group. Notably, these proteins were restored to levels comparable to those in the Sham KA+shMEF2A group (Fig. 6C). In contrast, the epilepsy-related excitatory receptor-associated proteins and transporters identified did not show a statistically significant difference among these three groups (Fig. S6G). To substantiate the observed alteration specificity, WB analysis was conducted on hippocampal lysates from Sham, KA-3d, KA+shMEF2A, and KA+Par groups. In alignment with proteomics data, KA-3d mice displayed significant reductions in Gabbr2, Gabra3, Gabrb2, and Gabrg2 expressions, partially ameliorated in the KA+shMEF2A and KA+Par groups but below the Sham control levels (Fig. 6D, E). These are consistent with our electrophysiological data (Fig. 5H), indicating that therapies targeting MEF2A may alleviate seizure severity and electrographic hyperexcitability by mitigating inhibitory synaptic protein loss.

Immunofluorescence staining revealed significantly reduced microglial MEF2A expression in KA+shMEF2A mice compared to KA control mice (Fig. 6F, G). Although parecoxib administration also decreased microglia MEF2A expression, the extent of reduction was less substantial than for AAV-shMEF2A treatment (Fig. 6F, G). Collectively, these indicate that microglial MEF2A downregulation mitigates KA-induced deficits in inhibitory synapse proteins, with therapeutic efficacy directly associated with MEF2A suppression.

### Targeting microglial MEF2A rescues seizure-induced GABAergic synapse dysfunction by inhibiting excessive microglial phagocytosis

**Fig. 7:**
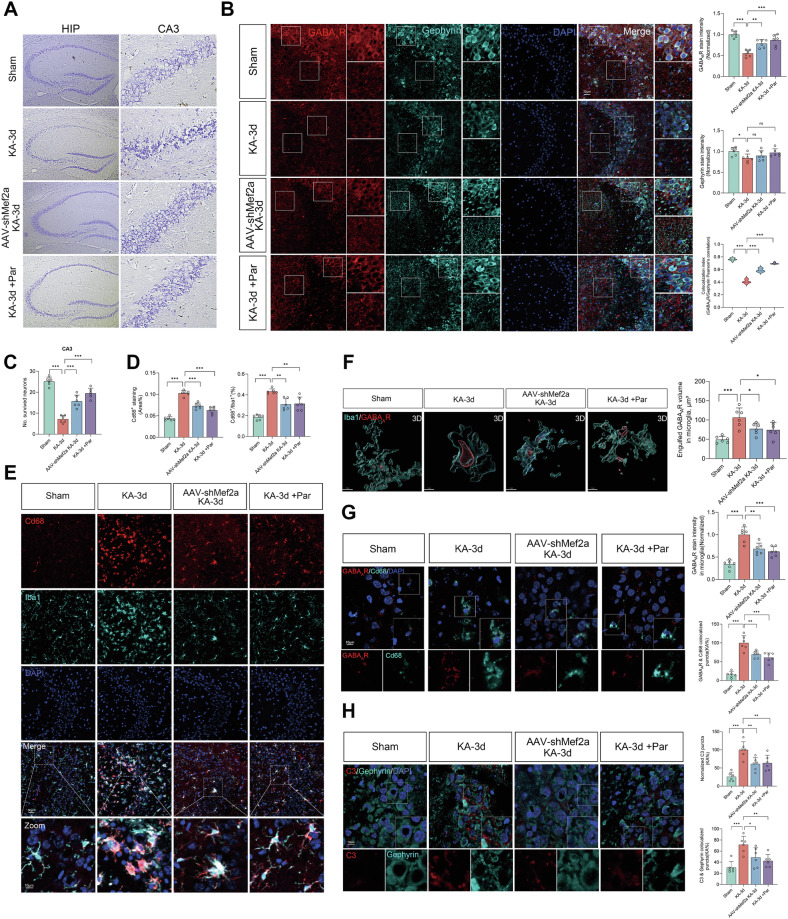
Targeting MEF2A treatment alleviates epilepsy-induced abnormalities in microglial phagocytosis of GABA receptors. **A** Representative Nissl staining images of the CA3 region in hippocampal sections from Sham, KA-3d, AAV- shMef2a KA-3d, and par+KA-3d groups (*n* = 6 per group). **B** Dual immunofluorescence staining for GABA_A_R (red) and Gephyrin (light blue) in hippocampal sections from Sham, KA-3d, AAV- shMef2a KA-3d, and Par+KA-3d groups; scale bar = 20 µm (*n* = 6 per group). Quantification of GABA_A_R expression intensity and Pearson’s correlation coefficient for GABA_A_R/Gephyrin co-localization; analyzed by one-way ANOVA; statistical comparisons between Sham and treatment groups. **C** Quantification of Nissl-stained positive cells in the CA3 region of hippocampal sections from A; analyzed by one-way ANOVA; ***p < 0.001 vs. Sham group. **D** Quantification of Cd68 expression intensity and Pearson’s correlation coefficient for Cd68/Iba1 co-localization in E; analyzed by one-way ANOVA; statistical comparisons between Sham and treatment groups. **E** Dual immunofluorescence staining for Cd68 (red) and Iba1 (cyan) in hippocampal sections from Sham, KA-3d, AAV- shMef2a KA-3d, and Par+KA-3d groups; scale bar = 20 µm (*n* = 6 per group). Quantification of CD68 expression intensity and Pearson’s correlation coefficient for Cd68/Iba1 co-localization; analyzed by one-way ANOVA; statistical comparisons between sham and treatment groups. **F** The double labeling of Iba1 (cyan) and GABA_A_R (red) illustrates the phagocytosis of inhibitory receptors by microglia within the hippocampal CA3 region, as demonstrated through 3D reconstructions (scale bars = 5 µm). Image quantification was conducted using a one-way ANOVA, with a sample size of *n* = 6 per group. **G** Dual immunofluorescence staining for Cd68 (cyan) and GABA_A_R (red) was performed on hippocampal CA3 sections from Sham, KA-3d, AAV-shMef2a KA-3d, and Par+KA-3d groups, with a scale bar of 10 µm (*n* = 6 per group). Quantification was analyzed using one-way ANOVA. **H** Dual immunofluorescence staining for Gephyrin (cyan) and C3 (red) was performed on hippocampal CA3 sections from Sham, KA-3d, AAV- shMef2a KA-3d, and Par+KA-3d groups, with a scale bar of 10 µm (*n* = 6 per group). Quantification was analyzed using one-way ANOVA. Data are presented as mean ± SD from each independent experiment unless stated otherwise. Statistical comparisons were performed using one-way ANOVA. Significance levels: *p < 0.05, **p < 0.01, ***p < 0.001 vs. indicated Sham or comparison groups.

Nissl staining showed that AAV-shMEF2A and parecoxib treatments have neuroprotective effects, as they significantly restored neuronal density in the CA3 region of the hippocampus in mice with KA-induced neuronal loss, compared to untreated controls (Fig. 7A, C). This suggests these treatments reduce epilepsy-related neuron damage. Further investigation was conducted to understand how targeting microglia aids in repairing epilepsy-induced damage. GABA_A_ receptors (GABA_A_Rs) serve as principal mediators of inhibitory neurotransmission within the mammalian CNS and play an essential role in maintaining synaptic inhibition [43]. GABA_A_Rs dysregulation is characteristic of epileptogenesis [44]. To examine the microglial MEF2A downregulation impact via AAV-shMEF2A, and parecoxib treatment on the integrity of inhibitory synapses during seizure, we conducted immunofluorescence co-labelling of GABAAR (Gabra1 in proteomics results) and its associated postsynaptic scaffold protein, gephyrin (GPHN), in hippocampal slices of KA-induced epileptic mice (KA-3d). Quantitative analyses demonstrated significantly reduced Gabra1 expression in KA-3d mice compared to that in Sham-operated controls (Fig. 7B, p < 0.001), with a pronounced GABA_A_R-GPHN colocalization decrease (Fig. 7B, p < 0.001). These suggest that epileptiform activity leads to GABA_A_Rs dissociation from synaptic sites, compromising inhibitory synapses stability. Importantly, administering either AAV-shMEF2A or parecoxib restored Gabra1 expression and significantly enhanced GABAAR and GPHN colocalization (Fig. 7B, *p* < 0.001 compared with KA-3d). This suggests that microglial MEF2A inhibition or parecoxib administration can mitigate seizure-induced synaptic receptor loss and re-establish synaptic receptor-scaffold interactions.

To elucidate the underlying mechanisms, we evaluated microglial phagocytic activity. Immunofluorescence analysis revealed that KA-3d mice exhibited a significantly higher number of CD68-positive microglia compared to sham controls (Fig. 7D, E, *p* < 0.001), with a 100% increase in CD68-IBA1 colocalization (Fig. 7D, E, *p* < 0.01), suggesting hyperactivation of microglial phagocytosis during acute seizures. Conversely, treatment with AAV-shMEF2A and parecoxib resulted in a reduction of CD68 expression (Fig. 7D, E, *p* < 0.01) and CD68-IBA1 colocalization (Fig. 7D, E, *p* < 0.05), approaching levels observed in Sham controls. These results indicate that downregulation of MEF2A or administration of parecoxib mitigates excessive microglial phagocytosis. To further investigate microglial subtypes, we performed dual immunofluorescence staining for CD68 and TREM2, a marker of disease-associated microglia (DAM). The results demonstrated that changes in TREM2 expression were consistent with those of CD68 across the four groups (Fig. S7A), further elucidating the subtype transformation of microglia during the acute phase of epilepsy and suggesting that targeting MEF2A may partially alleviate this transformation.

To investigate the potential role of aberrant synaptic phagocytosis in GABAergic impairment, we conducted a three-dimensional reconstruction of z-stack images obtained from IBA1/GABA_A_R dual immunofluorescence, alongside a co-localization analysis of GABA_A_R and CD68. Both methodologies demonstrated a significant increase in microglial phagocytosis of GABA_A_R in KA-3d mice compared to Sham controls, as indicated by a greater volume of internalized GABA_A_R puncta (*p* < 0.001) and a higher GABA_A_R -CD68 co-localization index (*p* < 0.01) (refer to Fig. 7F, G). Importantly, treatments with shMEF2A and parecoxib partially attenuated the microglial phagocytosis of GABA_A_R, thereby restoring the volume of phagocytosed GABA_A_R and co-localization levels closer to those observed in Sham controls (*p* < 0.05 compared to the KA group) (Fig. 7F, G). These results suggest that excessive microglial phagocytosis is a critical mechanism contributing to the acute-phase loss of GABA_A_R and reduced GPHN co-localization. In light of the established associations between aberrant synaptic phagocytosis and complement cascade activation [17], we further investigated the deposition of C3 (Complement component 3) on postsynaptic sites. Dual staining for C3 and GPHN revealed a significant increase in C3-GPHN co-localization in KA-3d mice (*p* < 0.001 compared to Sham), which was notably reduced by both interventions (*p* < 0.01 compared to KA) (Fig. 7H).

Collectively, these data demonstrate that the suppression of MEF2A hinders the transformation of microglia into a disease-associated phenotype (DAM), which in turn inhibits complement-dependent synaptic phagocytosis of GABA_A_R and mitigates seizure-associated hyperexcitability.

## Discussion

Glial cells’ important role in epilepsy pathogenesis has garnered increasing attention; however, mechanisms governing neuron-microglial interactions remain inadequately elucidated [7, 45, 46]. Thus, we identified MEF2A, a closely associated transcription factor of microglial state during acute epilepsy phase. Previous studies focused on the neuronal-enriched transcription factor MEF2A impact on neuronal pathology in various neurological disorders, including epilepsy; however, its functional role in microglia is insufficiently investigated [47], despite elevated MEF2A expression revealed in microglia derived from patients with Alzheimer’s disease [35, 48]. This study identified a significant upregulation of MEF2A in paraffin-embedded tissues derived from patients with TLE, which was correlated with the pathological transformation of microglia into an amoeboid phenotype. We selected our control group (patients undergoing emergency surgery ≤ 12 h post-trauma) based on evidence of incomplete microglial activation during this period [49, 50], supported by microglial morphology in our samples. Additionally, MEF2A expression was unchanged in mice 12 h post-TBI (Fig. S1D), confirming our comparative approach. In models of induced epileptiform activity, including primary neuron and microglia co-cultures and KA-induced TLE, microglial MEF2A expression was significantly increased, aligning with changes in microglial states. This indicates a direct relationship between neuronal activity and microglial MEF2A expression during epileptogenesis.

Following epileptic activity, damaged neurons release signaling molecules—especially ATP—which activates glial cells, thereby remodeling the microenvironment and exacerbating pathological changes [51]. Although ATP is a known mediator of neuroglial communication and microglial activation [46, 52, 53], our study shows that in epilepsy, ATP triggers a unique pathogenic microglial activation through MEF2A. We measured ATP levels in conditioned media from primary neurons and hippocampal tissues of C57 mice, three days after post-amygdala KA injection, and both exhibited significant elevation compared to controls—aligning with previous reports [22, 54–56]. Critically, in a transwell co-culture system comprising primary neurons and microglia, microglia exposed to epileptic neurons exhibited elevated activation markers and increased expression of MEF2A. This response was directly associated with exogenous ATP rather than LPS. Notably, the ATP-specific and LPS-insensitive induction of MEF2A was also observed in human HMC3 microglia, where ATP enhanced MEF2A expression while LPS did not. These results suggest that ATP specifically augments MEF2A-dependent pathogenic activation in microglia, highlighting MEF2A as a critical mediator of ATP-driven signaling pathways that connect neuronal excitability to microglial dysfunction in the context of epilepsy.

Building on existing evidence that Ca²⁺/CAMKII modulates MEF2A [40, 57], and considering our observation that epileptic neuronal activity results in elevated microglial Ca²⁺ levels—an increase comparable to that induced by exogenous ATP application—we concentrated our investigation on the Ca²⁺-associated ATP receptors, P2X4 and P2X7. The literature specifically highlights an upregulation of P2X7 expression in microglia following epileptic events [58, 59], a finding corroborated by our reanalysis of the single-cell sequencing data (Fig. S1F). It is noteworthy that P2X4 exhibits minimal expression, whereas P2X7 is highly expressed (Fig. S1E, F). Given the ATP-induced, P2X7-mediated Ca²⁺ influx [60, 61], we sought to elucidate the direct molecular link between P2X7 and MEF2A during the process of microglial activation. To functionally validate this mechanism, we demonstrated that antagonism of the P2X7 receptor suppresses intracellular Ca²⁺ elevation in HMC3 and BV2 microglial cells, thereby confirming P2X7 as the predominant receptor mediating ATP-induced Ca²⁺ signaling. WB analysis indicated that the inhibition of P2X7 receptors impeded the ATP-induced upregulation of MEF2A protein in epileptic contexts, thereby establishing a direct connection between ATP-P2X7-Ca²⁺ signaling and MEF2A expression. Functional assays conducted in BV2 cells revealed that the knockdown of MEF2A reduced microglial activation, whereas its overexpression enhanced activation in response to ATP stimulation. Collectively, these findings elucidate a pathological cascade wherein epileptic activity increases extracellular ATP levels, activating P2X7 receptors to facilitate Ca²⁺ influx. This process upregulates MEF2A expression, which subsequently promotes microglial activation, thus linking neuronal hyperexcitability to glial dysfunction in epilepsy.

Although HMC3 cells effectively validated the cross-species conservation of ATP-P2X7-Ca²⁺-MEF2A signaling, previous research indicates that their epigenetic defects resulting from SV40 immortalization compromise the integrity of inflammatory pathways. Consequently, downstream mechanisms were elucidated using BV2 models that maintain intact signaling networks.

As a pivotal transcription factor involved in the regulation of development, metabolism, and immunity, MEF2A facilitates the inflammatory activation of microglia through transcriptional reprogramming [62]. It is important to note that members of the MEF2 family exhibit a wide range of regulatory functions within microglial biology. Recent studies have shown that MEF2C inhibits microglial activation by maintaining p21-mediated RB stability, thereby preventing NFκB nuclear translocation[32]. Meanwhile, MEF2D enhances neuroprotection by inducing IL-10 to counteract TNF-α cytotoxicity [63] and mitigates ischemia-reperfusion injury through the suppression of TLR4/NFκB signaling[64]. In contrast, our findings suggest that MEF2A is a transcription factor closely associated with the activated phenotype of microglia. Bioinformatics analyses have predicted the binding of MEF2A to conserved motifs within the promoters of CD74 and NEK7, both of which are intimately associated with microglial activation. CD74 serves as a marker for microglial cell activation [65, 66], whereas NEK7 is a crucial regulatory factor for the activation of the NLRP3 inflammasome, which is closely linked to the progression of various neurodegenerative diseases [38, 67]. We have further validated these bindings through luciferase reporter assays and ChIP assays. In terms of functionality, the knockdown of MEF2A resulted in the suppression of ATP-induced expression of CD74/NEK7 and the formation of the NLRP3 inflammasome, whereas its overexpression enhanced these effects. Mechanistically, ATP initiates a Ca²⁺ influx via the P2X7 receptor, which activates the phosphorylation of CAMKII. Upon phosphorylation, CAMKII associates with HDAC5, prompting the nuclear export of HDAC5 and thereby ceasing the deacetylation of MEF2A. This process releases MEF2A from the repressive influence of HDAC5, facilitating its interaction with p300, which ultimately results in MEF2A hyperacetylation. This post-translational modification significantly augments both the DNA-binding affinity and the protein stability of MEF2A [40, 41, 68]. Thus, the ATP-Ca²⁺-CAMKII-HDAC5 pathway leads to MEF2A acetylation, activating CD74 and NEK7 to promote NLRP3 inflammasome assembly, positioning MEF2A as a key regulator of microglial pathogenic transformation.

Given the pivotal role of MEF2A in the aberrant activation of microglia, we investigated pharmacological agents capable of interacting with this protein. Drawing on structural insights from previous studies on MEF family inhibitors, we identified parecoxib, a NSAID, as a potential inhibitor of MEF2A. Although parecoxib is known to attenuate general neuroinflammation in preclinical models, its effects on microglial activation have been less thoroughly examined [69–71]. In this study, we were the first to confirm its inhibitory effect on MEF2A in vitro, indicating that targeting MEF2A with parecoxib may represent a novel therapeutic strategy for epilepsy. Besides, celecoxib, an NSAID that does not inhibit MEF2A, demonstrated a minimal therapeutic effect in epileptic mice. These findings underscore the potential of parecoxib as a safe anti-epileptic intervention, attributed to its novel mechanism of action involving the suppression of MEF2A.

In vivo, although AAV-shMEF2A knockdown and intracranial parecoxib did not significantly delay the onset of seizures in TLE mouse models, both effectively reduced long-term seizure frequency, altered EEG patterns, and decreased microglial activation and neuroinflammation. Notably, the sustained efficacy observed following a single-dose administration of parecoxib underscores the critical importance of the early therapeutic window. This observation suggests that inhibiting the initial acute inflammatory response plays a significant role in mitigating the subsequent pathological processes associated with epilepsy. During the chronic phase, these treatments also improved epilepsy-related comorbidities such as anxiety, depression, and cognitive impairment, which aligns with supporting previous findings on the detrimental impact of excessive microglial activation on neurons [72–74]. These results collectively establish a proof-of-concept for MEF2A-targeted epilepsy therapy. Furthermore, the reduction in microglial hyperactivation supports existing evidence that neuroimmune modulation is associated with the sustained suppression of recurrent seizures and the mitigation of epileptic comorbidities [75, 76].

In examining the specific mechanisms underlying the in vivo therapeutic effects, an analysis of AAV-shMEF2A-treated mice with acute epilepsy demonstrated that the suppression of MEF2A alleviates the loss of inhibitory synaptic receptors, a critical pathological feature of epilepsy [44, 77, 78]. AAV-shMEF2A and parecoxib therapies exhibited comparable efficacy in mitigating the downregulation of inhibitory synapses by reducing the expression of CD68 and TREM2, which are closely linked to the phagocytic function of microglia [79]. In addition to suppressing the phenotypic transition of microglia, we directly observed microglia actively phagocytosing GABA_A_R receptors in epileptic mice, and this phagocytic activity was significantly diminished after treatment. This reduction was associated with increased GABA_A_R density and enhanced co-localization with GPHN, the essential scaffold protein for inhibitory synapses. The quantitative analysis revealed an inverse correlation between CD68 expression levels and GABA_A_R recovery, indicating that hyperactive microglia exacerbate the loss of postsynaptic GABAergic receptors—a mechanism previously implicated in epileptogenesis [16, 72, 73]. These data provide direct evidence that MEF2A-targeted therapy specifically modulates microglia-mediated phagocytosis of inhibitory receptors following epileptogenesis. A pivotal study has notably identified an upregulation of CD74 in microglia that selectively engage in the phagocytosis of inhibitory receptors [17]. When integrated with our findings, this evidence collectively suggests that CD74 functions downstream of MEF2A in the activation of pathogenic microglia, thereby independently corroborating the validity of our targeting approach. Furthermore, both interventions were observed to decrease the deposition of complement C3, a crucial opsonin involved in marking inhibitory synapses for microglial phagocytosis. This reduction likely disrupts the recognition signal necessary for synaptic elimination [80, 81], thereby providing a comprehensive explanation for the observed decrease in the phagocytosis phenomenon. Collectively, our study demonstrates that MEF2A inhibition mitigates the progression of epilepsy by curtailing aberrant microglial phagocytosis of inhibitory synapses, thereby alleviating the progressively exacerbated excitatory/inhibitory imbalance characteristic of chronic epilepsy in mice.

Our study elucidates a neuron-to-microglia signaling pathway in which activity-dependent neuronal ATP release following epileptic events activates microglial P2X7 receptors. This activation initiates a specific Ca²⁺/CAMKII/HDAC5 signaling cascade, ultimately leading to the activation of the transcription factor MEF2A. Importantly, we demonstrate that MEF2A directly regulates the transcription of NEK7, a protein involved in inflammasome assembly, and CD74, which enhances the microglial phagocytosis of inhibitory synapses. This work provides the first direct evidence that MEF2A acts as a key molecular node connecting aberrant neuronal discharge to microglial synaptic engulfment via an integrated signaling cascade. Notably, we identify the clinically available NSAID parecoxib as an effective inhibitor of MEF2A. Intracranial administration of parecoxib replicates the therapeutic effects of microglia-specific MEF2A knockdown, mitigating seizure-associated pathology and preserving inhibitory synapses independently of traditional AEDs. These findings establish pharmacological MEF2A inhibition as a viable strategy for targeting the core excitatory/inhibitory imbalance in epilepsy.

The study possesses limitations that merit careful consideration. As a preclinical investigation, the therapeutic implications necessitate rigorous validation in clinical settings. Although intracranial drug delivery poses translational challenges, our focus on drug-resistant TLE—a condition often managed through surgical intervention—may offer an opportunity for intraoperative pharmacological application during resection procedures. Mechanistically, we show that ATP-P2X7-Ca²⁺ activates MEF2A in microglia, but the full regulatory role of MEF2A is not yet fully understood. MEF2A might undergo post-translational modifications like SUMOylation, methylation, or phosphorylation after epileptic events, but their presence and effects in epilepsy need confirmation. MEF2A could also affect neuroinflammation and microglial activation through genes related to mitochondrial function and stress responses, though these connections are still speculative. Importantly, our approach, to develop adjunctive therapies that complement conventional AEDs, targets microglia. Although the current model did not achieve complete seizure suppression, microglial modulation specifically targets the fundamental excitatory/inhibitory imbalance resulting from its abnormal phagocytosis, rather than serving as a replacement for standard AEDs. Future research will investigate the potential benefits of combining MEF2A-targeted strategies with existing AED treatments.

## Materials and methods

### Animals

Six-week-old male C57BL/6 J wild-type mice (20–25 g) were obtained from Beijing Sibeifu Biotechnology, whereas male C57BL/6JGpt-Cx3cr1-iCre transgenic mice of similar age and weight were obtained from Jiangsu GemPharmatech. Animals were allocated to experimental groups through the use of computer-generated random sequences. Given the significant mortality risk associated with the induction of status epilepticus (SE), all experimental procedures, including drug administration and behavioral assessments, were conducted without the implementation of blinding. The experiments complied with the NIH Guide for the Care and Use of Laboratory Animals and were approved by the Tianjin Huanhu Hospital Ethics Committee (approval number: HHLL-2025-044).

### Human brain tissue

Six temporal lobectomy samples were collected from patients with refractory TLE, and six control samples were from TBI patients without epilepsy. The study adhered to the Declaration of Helsinki and was approved by the Shengjing Hospital Ethics Committee (Approval Number: 2020PS787K); informed consent was obtained from all subjects. Detailed information of patients is in Table S1.

### scRNA seq and RNA-seq data analyses

Human scRNA-seq data (GSE190452) was processed with Seurat, using Combat correction and UMAP for visualization, keeping cells with > 200 genes and < 10% MT reads. Microglial DEGs were identified using Wilcoxon (p < 0.05, log2FC > 0.5). Mouse bulk RNA-seq (GSE99577; 1/2/4/12 dpi) time-series expression was correlated with human microglial DEGs via Spearman’s method.

### ATP assay

Extracellular ATP levels were measured in primary neuronal cultures after 3 h of Mg²⁺-free exposure and in bilateral hippocampal tissues on the third day post-surgery using a Beyotime ATP Assay Kit (S0027). Luminescence was recorded following a 3–5-minute incubation with detection reagent at room temperature, and tissue ATP levels were normalized to protein content (nmol/mg protein).

### Primary neuron culture and establishment of epileptiform discharge model

Primary hippocampal neurons were isolated from mice at gestational day 18 and cultured following established protocols, allowing for a maturation period of 7 to 10 days. Epileptiform discharges were induced by incubating the neurons in a magnesium-free extracellular solution composed of 145 mM NaCl, 2.5 mM KCl, 2 mM CaCl₂, 10 mM HEPES/glucose, and 0.002 mM glycine, with a pH of 7.2 and an osmolarity of 325 mOsm, at 37°C for a duration of 3 h.

### Extraction of primary microglia and neuron-microglia co-culture system

Primary microglia were isolated from postnatal day 0 (P0) mice utilizing the shake-off method, conducted at 250 revolutions per min for a duration of 2 h, in accordance with established protocols [22]. Neurons, subjected to treatment with magnesium-free medium for 3 h, were subsequently co-cultured with microglia at a neuron-to-microglia ratio of 1:2. This co-culture was performed using Transwell inserts with a pore size of 0.4 μm, maintained for 24 h at 37°C in an atmosphere containing 5% CO₂.

### Cell lines and cell culture

BV2 microglial, HEK293T, and HMC3 cells, sourced from Procell Life Technology Co., Ltd. (Wuhan, China), were cultured for the experiments. BV2 and HEK293T cells used DMEM, and HMC3 cells used MEM, both supplemented with 10% FBS and 1% antibiotics. Cultures were incubated at 37°C with 5% CO₂.

### Intracellular calcium level assay

Cells in confocal dishes were incubated with 2 μM Fluo-4 AM in PBS for 30 min at 37°C in the dark, washed, allowed 20 min for de-esterification at 37°C, and then fluorescence was measured using laser-scanning confocal microscopy.

### Immunofluorescence and image analysis

Cells and brain sections were immunostained using primary antibodies for MEF2A, IBA1, MAP2, NeuN, iNOS, GABAAR, GPHN, C3, TREM2 and CD68, then imaged with confocal microscopy. Co-staining pairs MEF2A/IBA1, GABAAR/GPHN, IBA1/GABAAR, GABAAR/CD68, C3/GPHN, CD68/TREM2 and CD68/IBA1were used for quantitative immunofluorescence, immunocytochemistry (ICC), 3D reconstruction and Sholl analyses. The details of all antibodies are in Table S2.

### qRT-PCR

RNA was extracted with TRIzol reagent (Solarbio, Beijing, China). cDNA synthesis and qPCR were conducted using a One-Step TB Green PrimeScript RT-PCR Kit (Takara Bio, Japan, Cat. No. RR066A) as per the manufacturer’s guidelines. The 7500 Fast RT-PCR System (Applied Biosystems) was used for analysis. Primers, sourced from Sangon Biotech (Shanghai, China), are listed in Table S3.

### Protein extraction and WB

Proteins were extracted using RIPA buffer with inhibitors, quantified via BCA assay, separated on 12.5% SDS-PAGE, and transferred to PVDF membranes for WB analysis. Membranes were blocked, incubated with primary and HRP-conjugated secondary antibodies (Table S2), detected by ECL, and quantified using ImageJ, normalized to β-actin or GAPDH. Full-length uncropped western blots are provided in the Supplementary Information.

### Luciferase assay and ChIP assay

Luciferase assays were employed to assess the promoter activity of NEK7 and CD74 (refer to Table S4 for constructs) in HEK293T cells transfected with MEF2A, utilizing Renilla luciferase for normalization. ChIP was conducted using MEF2A-specific antibodies, with histone H3 and IgG serving as controls, to evaluate binding enrichment at the NEK7 and CD74 promoters (see Table S5 for primer sequences). This process adhered to standard protocols involving crosslinking, sonication, immunoprecipitation, and PCR detection.

### Lentivirus transfection for stable cell line construction

Stable MEF2A knock-down and overexpression BV2 cell lines were created using lentiviral transduction with BrainVTA vectors (Table S6) at MOI 25, then selected with puromycin and verified for MEF2A expression via qRT-PCR, PCR, and WB.

### Co-IP assay

Protein-protein interactions (MEF2A–HDAC5, MEF2A–p300, HDAC5–CAMKII) were examined through co-immunoprecipitation using antibodies against HDAC5, CAMKII, or p300, alongside IgG controls. The resulting complexes from cell lysates were analyzed by WB, with antibody specifics in Table S3.

### Molecular docking

The MEF2A protein structure from the Protein Data Bank and parecoxib’s chemical structure from PubChem were preprocessed with hydrogen atoms and charge calculations. AutoDock Vina performed molecular docking to evaluate binding affinity and energy-minimized structures. PyMOL visualized the interactions and binding modes of the MEF2A-parecoxib complex.

### Adeno-associated virus transduction

Stereotaxic bilateral microinjections of rAAV constructs with MEF2A-targeting shRNA or an empty vector control (BrainVTA; AAV2/MG1.2, ≥2×10¹² vg/mL; Table S7) were administered into the hippocampal CA3 (250 nL/hemisphere) of anesthetized Cx3cr1-iCre mice. Analyses were conducted 3 weeks later.

### Mouse model of epilepsy with LFP recording

The KA amygdala epilepsy model was developed through the stereotaxic microinjection of kainic acid into the right basolateral amygdala of anesthetized mice, which were implanted with hippocampal electrodes. The Racine scale is utilized to evaluate the severity of epileptic seizures in murine models. On the 7th day following KA injection, continuous LFP recordings were conducted on awake mice for a period of two hours. Seizure-like events (SLEs) were defined as spontaneous bursts with a frequency over 5 Hz, amplitude more than double the baseline, and lasting longer than 5 s.

### Drug administration

A-438079, a P2X7 receptor antagonist, was dissolved in DMSO to make a 10 mM stock solution and then diluted with PBS to 20 μM for cell experiments. Parecoxib was prepared as a 10 mg/ml stock in PBS and diluted to 5 mg/mL with saline. For intracerebroventricular administration, mice received 100 μM of Parecoxib per side in 2 μl through a guide cannula 1 hour before KA injection.

### Mice behavior tests

Behavioral assessments commenced on the seventh post-operative day, incorporating the Morris Water Maze to evaluate escape latency, the Novel Object Recognition Test to determine the discrimination ratio, the sucrose preference test to measure the percentage of sucrose preference, and the open field test to assess total distance traveled and time spent in the center. Additionally, the Y-maze test, which evaluates spontaneous alternation percentage and total arm entries, was administered on the twenty-first day.

### Proteomics

A proteomic analysis utilizing Data-Independent Acquisition Mass Spectrometry (DIA-MS) on twelve hippocampal samples identified proteins with significant alterations, employing a fold-change threshold greater than 1.5 and a *p*-value less than 0.05.

### Nissl staining

A Nissl staining kit (Beyotime, C0117, Jiangsu, China) was used as per instructions. Stained cells appeared speckled purple and were examined under a Nikon Eclipse 80i microscope to assess neuronal integrity by counting Nissl-positive neurons.

### Statistical analysis

Statistical analyses used Student’s t-test for two groups and one-way/two-way ANOVA for multiple groups, with Levene’s test confirming variance homogeneity (*p* > 0.05). For unequal variances (*p* < 0.05), Welch’s t-test or Games-Howell tests were used. Non-parametric tests (Mann-Whitney U, Kruskal-Wallis) were applied if the data didn’t meet normality. Significance was set at *p* < 0.05.

## Supplementary information

supplementary materials

Original Western Blots.

Dataset 1

## Data Availability

Further requests for materials in this study should be directed to the lead contact, Dr Hua Yan: yanh2023@tmu.edu.cn. This paper does not report original code or original scRNA-seq/RNA-seq data. Any additional information required to reanalyze the data reported in this paper is available from the lead contact upon request.
