# Supplementary material for: Targeting MEF2A suppresses microglial hyperactivation and synaptic phagocytosis to attenuate epilepsy pathogenesis
Source: Cell Death Dis. 2026 May 22;17(1):645. doi: 10.1038/s41419-026-08860-5 (PMC13377041; doi:10.1038/s41419-026-08860-5)
Supplement: Supplementary file 1 — supplementary materials [file 41419_2026_8860_MOESM1_ESM.docx]

**Table S1**: The clinical characterization of TLE patients and TBI patients.

| Sex | Age  (years) | Duration  (years) | Preoperative ASM  consumption | Side of resected temporal lobe | Pathological  diagnosis |
| --- | --- | --- | --- | --- | --- |
| F | 63 | 13 | OXC‌ | R | NL, G |
| M | 38 | 9 | OXC‌, LTG | L | NL, G |
| F | 49 | 15 | OXC‌, LTG | R | NL, G |
| F | 46 | 12 | VPA, LTG | L | NL, G |
| M | 30 | 4 | CBZ, TPM | L | NL, G |
| F | 45 | 10 | CBZ | R | NL, G |
| F | 40 | 0 | None | L | N |
| F | 44 | 0 | None | R | N |
| M | 36 | 0 | None | L | N |
| M | 53 | 0 | None | R | N |
| F | 64 | 0 | None | R | N |
| F | 56 | 0 | None | R | N |

ASM, anti-seizure medication; E, epilepsy; C, control; M, male; F, female; CBZ, carbamazepine; TPM, topiramate; OXC‌, Oxcarbazepine; LTG, lamotrigine; VPA, Valproic Acid; L, left; R, right; NL, neuron loss; G, gliosis; N, relative normal.

**Table S2**: Specific information for antibodies.

| **Antibodies** | **Applications** | | **Source** | **Identifier** |
| --- | --- | --- | --- | --- |
| MEF2A mAb | | WB: 1:1000; IF: 1:100; COIP: 2 µg per 500 µg | Santa Cruz (CA, USA) | Cat# sc-17785 |
| IBA1 pAb | | IF: 1:250 | proteintech(Wuhan, China) | Cat# 10904-1-AP |
| IBA1 mAb | | IF: 1:200 | CST (USA) | Cat# 17198 |
| CD74 mAb | | WB: 1:1000 | Abcam (Cambridge, UK) | Cat# ab289885 |
| NEK7 pAb | | WB: 1:2500 | proteintech(Wuhan, China) | Cat# 31422-1-AP |
| NLRP3 mAb | | WB: 1:2000 | proteintech(Wuhan, China) | Cat# 68102-1-Ig |
| MEF2A pAb | | IHC: 1:250; WB: 1:1000 | proteintech(Wuhan, China) | Cat# 28819-1-AP |
| iNOS mAb | | IF: 1/500 | Abcam (Cambridge, UK) | Cat# ab178945 |
| HDAC5 mAb | | WB: 1:1000; COIP: 1:200 | CST (USA) | Cat# 98329 |
| GAPDH mAb | | WB: 1:80000 | proteintech(Wuhan, China) | Cat# 60004-1-Ig |
| Beta Actin mAb | | WB: 1:20000 | proteintech(Wuhan, China) | Cat# 66009-1-Ig |
| CaMKIIαmAb | | WB: 1:500; COIP: 2 µg per 500 µg | Santa Cruz (CA, USA) | Cat# sc-13141 |
| p-CaMKII mAb | | WB: 1:500 | Santa Cruz (CA, USA) | Cat# sc-32289 |
| Lamin B1 mAb | | WB: 1:80000 | proteintech(Wuhan, China) | Cat# 66095-1-Ig |
| p300 mAb | | COIP: 1/30; WB: 1:1000 | Abcam (Cambridge, UK) | Cat# ab275378 |
| GABAA Ra mAb | | IF: 1:200 | Santa Cruz (CA, USA) | Cat# sc-376282 |
| GABRA3 pAb | | WB: 1:1000 | proteintech(Wuhan, China) | Cat# 12708-1-AP |
| GABRG2 pAb | | WB: 1:1000 | proteintech(Wuhan, China) | Cat# 14104-1-AP |
| GABA B2 mAb | | WB: 1:1000 | HuaAnBio(Hangzhou, China) | Cat# ET7106-88 |
| GABRB2 pAb | | WB: 1:1000 | HuaAnBio(Hangzhou, China) | Cat# HA500289 |
| CD68 mAb | | IF: 5 µg/mL | (Invitrogen, USA) | Cat# 14-0681-82 |
| Gephyrin pAb | | IF: 1:100 | proteintech(Wuhan, China) | Cat# 12681-1-AP |
| NeuN mAb | | IF: 1:100 | proteintech(Wuhan, China) | Cat# 66836-1-Ig |
| MAP2 pAb | | IF: 1:100 | proteintech(Wuhan, China) | Cat# 17490-1-AP |
| CD74 mAb | | WB: 1:2000 | proteintech(Wuhan, China) | Cat# 66390-1-Ig |
| C3 mAb | | IF: 1:200 | Santa Cruz (CA, USA) | Cat# sc-28294 |
| TREM2 mAb | | IF: 1:100 | Abcam (Cambridge, UK) | Cat# ab305103 |
| Alexa Fluor™ 555 Goat anti-Rabbit IgG (H+L) | | IF: 1:500 | (Invitrogen, USA) | Cat# A-31572 |
| Alexa Fluor™ 555 Goat anti-Mouse IgG (H+L) | | IF: 1:500 | (Invitrogen, USA) | Cat# A-31570 |
| Alexa Fluor™ 647 Goat anti-Rabbit IgG (H+L) | | IF: 1:500 | (Invitrogen, USA) | Cat# A-31573 |
| Alexa Fluor™ 647 Goat anti- Mouse IgG (H+L) | | IF: 1:500 | (Invitrogen, USA) | Cat# A-31571 |

IF, Immunofluorescence; WB, Western blotting; IHC: Immunohistochemistry; COIP: Co-Immunoprecipitation.

**Table S3**: List of qPCR detection primers.

| Primer name | Source |
| --- | --- |
| qMef2a primer-F: 5' ACCGGCAGTGCAAGTGGGATGTT 3' | Tsingke Biotechnology Co., Ltd (Beijing, China) |
| qMef2a primer-R: 5' GCGGGGGAGACTTTGTAGGCATGA 3' | Tsingke Biotechnology Co., Ltd (Beijing, China) |
| qmGAPDH primer-F: 5’GCCCATCACCATCTTCCAGGAGCG 3’ | Tsingke Biotechnology Co., Ltd (Beijing, China) |
| qmGAPDH primer-R: 5’GCAGAAGGGGCGGAGATGATGACC 3’ | Tsingke Biotechnology Co., Ltd (Beijing, China) |
| qmCCL2 primer-F: 5' TTAAAAACCTGGATCGGAACCAA 3' | Sangon, Shanghai, China |
| qmCCL2 primer-R: 5' GCATTAGCTTCAGATTTACGGGT 3' | Sangon, Shanghai, China |
| qmiNOS primer-F: 5' GTTCTCAGCCCAACAATACAAGA 3' | Sangon, Shanghai, China |
| qmiNOS primer-R: 5' GTGGACGGGTCGATGTCAC 3' | Sangon, Shanghai, China |
| qmIL1B primer-F: 5' GCAACTGTTCCTGAACTCAACT 3' | Sangon, Shanghai, China |
| qmIL1B primer-R: 5' ATCTTTTGGGGTCCGTCAACT 3' | Sangon, Shanghai, China |
| qmIL6 primer-F: 5' TAGTCCTTCCTACCCCAATTTCC 3' | Sangon, Shanghai, China |
| qmIL6 primer-R: 5' TTGGTCCTTAGCCACTCCTTC 3' | Sangon, Shanghai, China |
| qMEF2A primer-F: 5' GGTCTGCCACCTCAGAACTTT 3' | Sangon, Shanghai, China |
| qMEF2A primer-R: 5' CCCTGGGTTAGTGTAGGACAA 3' | Sangon, Shanghai, China |
| qNEK7 primer-F: 5' GCCTTACGACCGGATATGGG 3' | Sangon, Shanghai, China |
| qNEK7 primer-R: 5' CACTAAATTGTCCGCGACCAA 3' | Sangon, Shanghai, China |
| qCD74 primer-F: 5' GACGAGAACGGCAACTATCTG 3' | Sangon, Shanghai, China |
| qCD74 primer-R: 5' GTTGGGGAAGACACACCAGC 3' | Sangon, Shanghai, China |
| qGAPDH primer-F: 5' CTTTTGCGTCGCCAG 3' | Sangon, Shanghai, China |
| qGAPDH primer-R: 5' TTGATGGCAATATCCAC 3' | Sangon, Shanghai, China |

**Table S4**: Plasmid-related information.

| name | vector | Source |
| --- | --- | --- |
| h-MEF2A | pHBLV-CMV-MCS-3FLAG-EF1-ZsGreen-T2A-PURO | Hanheng Biological, Beijing, China. |
| h-NEK7 | pGL3-Basic | Hanheng Biological, Beijing, China. |
| h-NEK7 s | pGL3-Basic | Hanheng Biological, Beijing, China. |
| h-CD74 | pGL3-Basic | Hanheng Biological, Beijing, China. |
| h-CD74 s | pGL3-Basic | Hanheng Biological, Beijing, China. |

To obtain the details of the plasmid, please contact the corresponding author via email to request it.

**Table S5**: Primer sequences for qPCR of CHIP Assay

| Primer name | Source |
| --- | --- |
| qMEF2A primer-F: 5' GGTCTGCCACCTCAGAACTTT 3' | Sangon, Shanghai, China |
| qMEF2A primer-R: 5' CCCTGGGTTAGTGTAGGACAA 3' | Sangon, Shanghai, China |
| qNEK7 primer-F: 5' GCCTTACGACCGGATATGGG 3' | Sangon, Shanghai, China |
| qNEK7 primer-R: 5' CACTAAATTGTCCGCGACCAA 3' | Sangon, Shanghai, China |
| qCD74 primer-F: 5' GACGAGAACGGCAACTATCTG 3' | Sangon, Shanghai, China |
| qCD74 primer-R: 5' GTTGGGGAAGACACACCAGC 3' | Sangon, Shanghai, China |
| qGAPDH primer-F: 5' CTTTTGCGTCGCCAG 3' | Sangon, Shanghai, China |
| qGAPDH primer-R: 5' TTGATGGCAATATCCAC 3' | Sangon, Shanghai, China |

**Table S6**: Lentivirus information

|  | name | virus titer | Source |
| --- | --- | --- | --- |
| Overexpression of Mef2a | Experimental: LV-CMV-Mef2a-P2A-mCherry-PGK-Puro-WPRE;  Control: LV-0974 LV-CMV-mCherry-PGK-Puro-WPRE. | ≥2.00E+8 TU/ml | BrainVTA, Wuhan, China |
| Knockdown of Mef2a | Experimental: LV-U6-shRNA(Mef2a)-CMV-mcherry-T2A-Puro-WPRE;  Control: LV-0144，LV-U6-shRNA(scramble)-CMV-mcherry-T2A-Puro-WPRE. | ≥2.00E+8 TU/ml | BrainVTA, Wuhan, China |

To obtain the viral interference sequence, please contact the corresponding author via email to request it.

**Table S7**: Adeno-Associated Virus information

|  | name | serotype | virus titer | Source |
| --- | --- | --- | --- | --- |
| Construction of viral vectors | rAAV-CX3CR1-DIO-EGFP-5' miR30-shRNA(Mef2a)-3'miR30-WPREs |  |  |  |
| Knockdown of Mef2a | rAAV-CX3CR1-DIO-EGFP-5' miR30-shRNA(Mef2a)-3'miR30-WPREs | AAV2/MG1.2 | ≥2.00E+12vg/ml | BrainVTA, Wuhan, China |
| vector of Mef2a | rAAV-CX3CR1-DIO-EGFP-5' miR30-shRNA(empty)-3'miR30-WPREs | AAV2/MG1.2 | ≥2.00E+12vg/ml | BrainVTA, Wuhan, China |

To obtain the viral interference sequence, please contact the corresponding author via email to request it.

**Table S8**: DIA Proteomics Sample Testing Report

Protein determination scale

| NO | Sample Name | Group | Volume after sample extraction  （μL） | Protein concentration  （μg/μL） |
| --- | --- | --- | --- | --- |
| 1 | con1 | Crtl | 200 | 4.72 |
| 2 | con2 |  | 200 | 3.18 |
| 3 | con3 |  | 200 | 5.43 |
| 4 | con4 |  | 200 | 4.67 |
| 5 | epi3d1 | KA-3d | 200 | 2.79 |
| 6 | epi3d2 |  | 200 | 1.63 |
| 7 | epi3d3 |  | 200 | 3.95 |
| 8 | epi3d4 |  | 200 | 3.02 |
| 9 | shME1 | shMef2a KA-3d | 200 | 6.89 |
| 10 | shME2 |  | 200 | 3.83 |
| 11 | shME3 |  | 200 | 4.20 |
| 12 | shME4 |  | 200 | 5.86 |


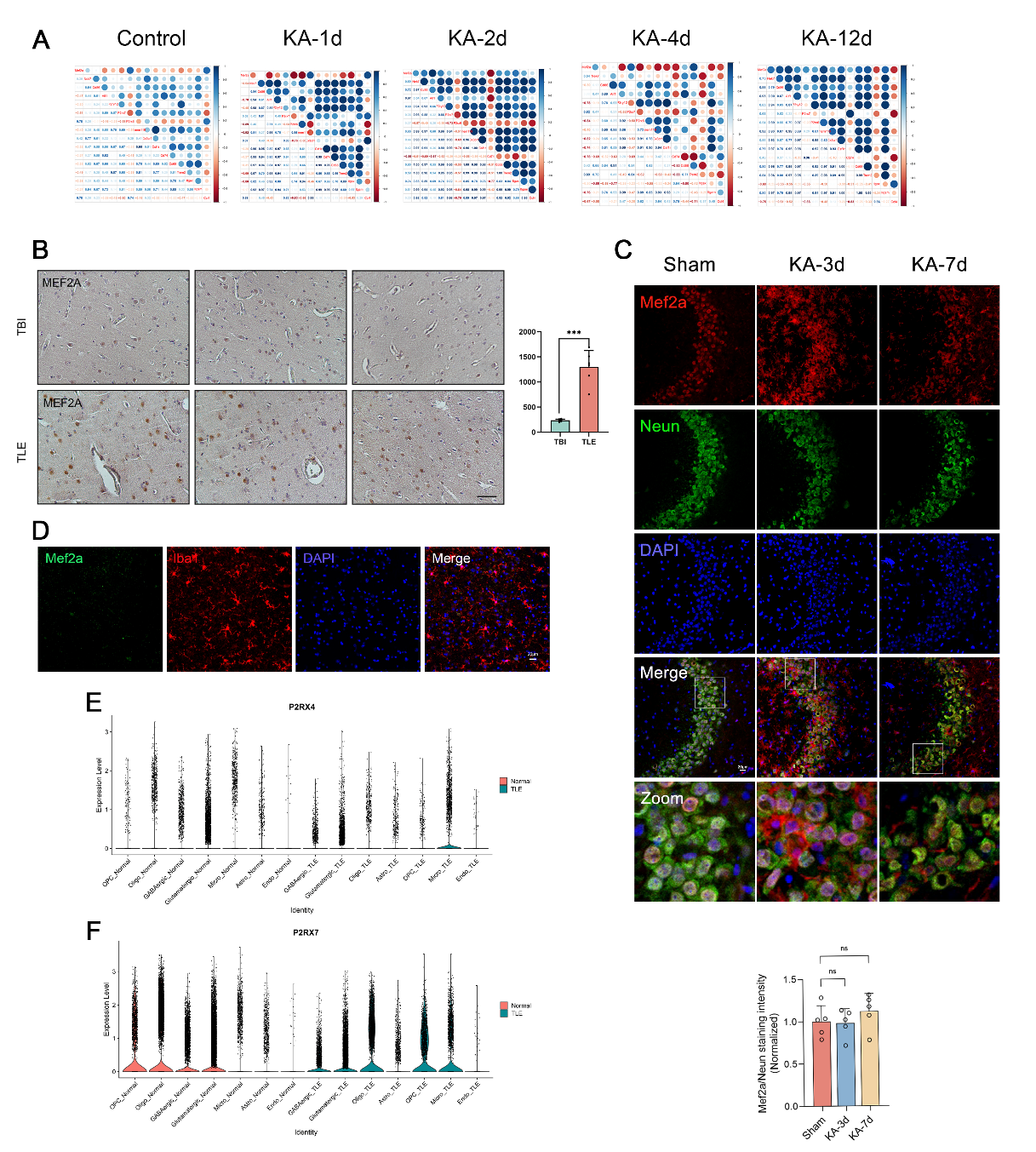


**Figure S1: Characterization of MEF2A/Mef2a expression profiles and purinergic receptor distribution.** (A) Correlation analysis between Mef2a and canonical microglial marker genes derived from Mouse RNA-seq data; (B) Representative immunohistochemical staining demonstrating increased MEF2A expression in human epileptogenic foci compared to trauma controls (n = 6 per group, ****p* < 0.001). Scale bar represents 100 μm; (C) Double immunofluorescence staining for Mef2a (red) and the neuronal marker NeuN (green). Scale bar represents 20 μm. The quantification illustrates the manual analysis of Mef2a expression intensity specifically within NeuN-positive neurons, calculated as the Mef2a/NeuN ratio in co-localized cells. 'ns' denotes no significant difference (p > 0.05); (D) Representative double immunofluorescence images of Iba1 (red) and Mef2a (light blue) in the traumatic brain injury (TBI) model at 12 hours post-injury. Scale bar represents 20 µm; (E–F) Violin plots (VlnPlot) depicting the expression levels of P2rx4 (E) and P2rx7 (F) within epilepsy-associated microglial clusters. Data Presentation: The data are expressed as mean ± standard deviation (SD). Statistical analyses were conducted using an unpaired two-tailed Student’s t-test for comparisons between two groups, or a one-way analysis of variance (ANOVA) for comparisons involving multiple groups. KA refers to the post-intra-amygdala stereotaxic microinjection of kainic acid.


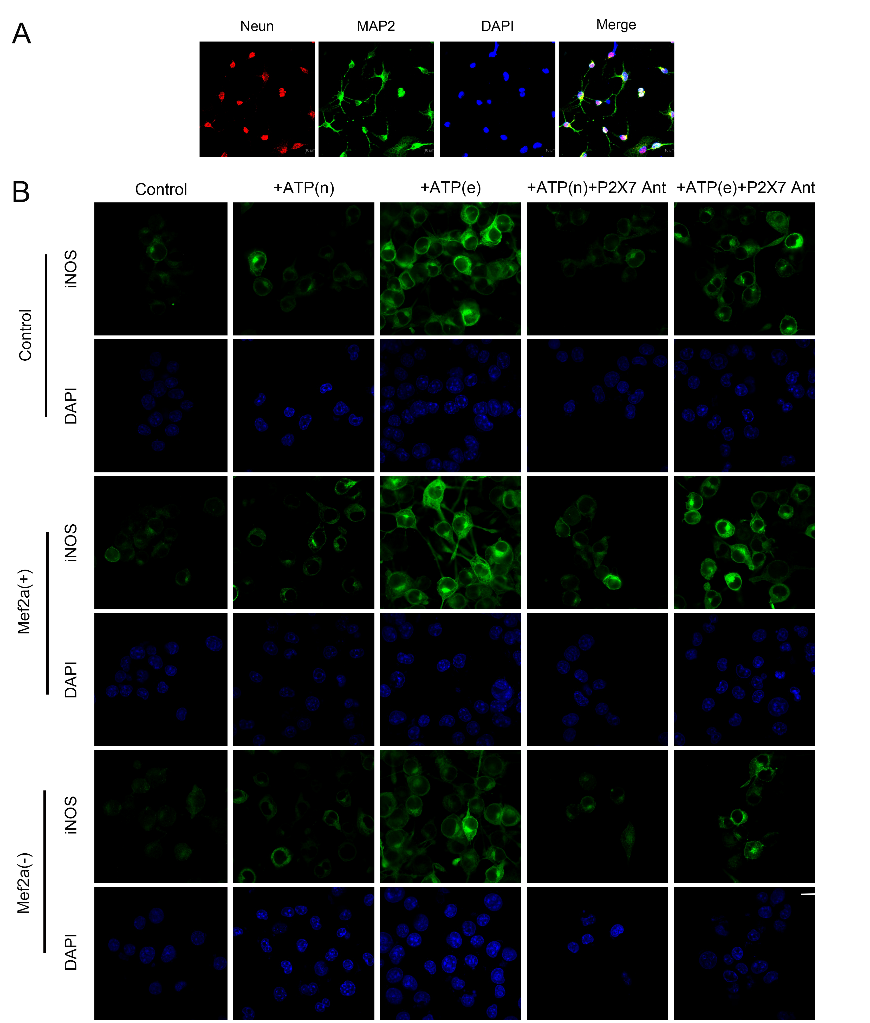


**Figure S2** (A) Primary mature neurons were identified by immunofluorescence staining for neuronal markers (Neun/MAP2) (B)The single-channel diagram of Fig3E, immunofluorescence images of INOS (green) and DAPI in control, Mef2a(+), Mef2a(-) BV2 cell, scale bar = 20μm,


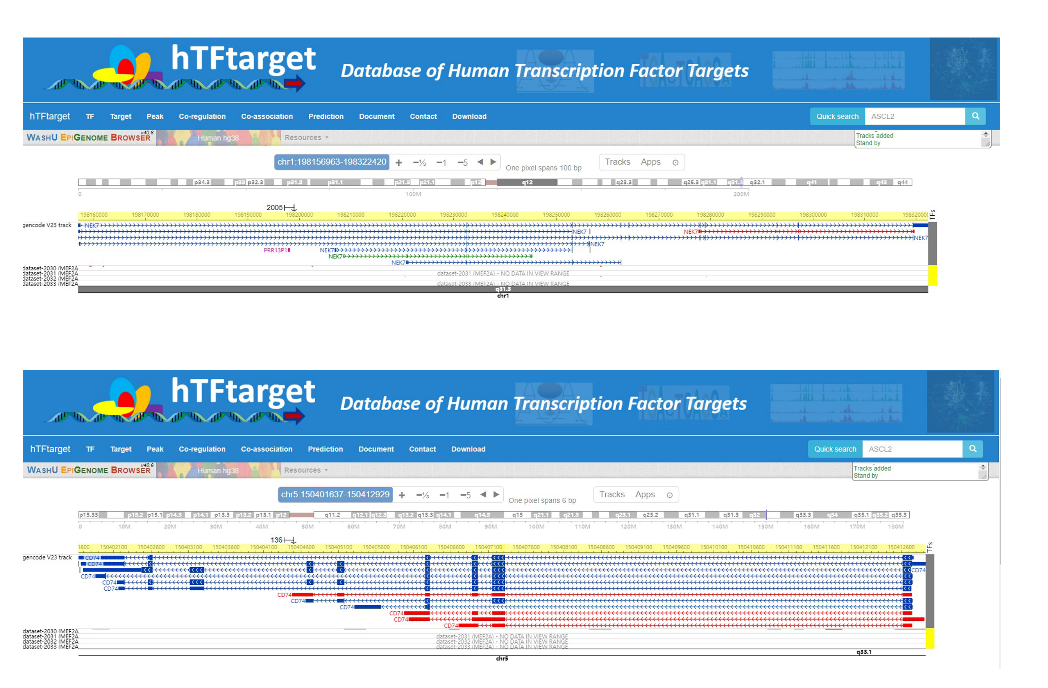


**Figure S3** The identification of target genes for MEF2A within the hTFtarget database.


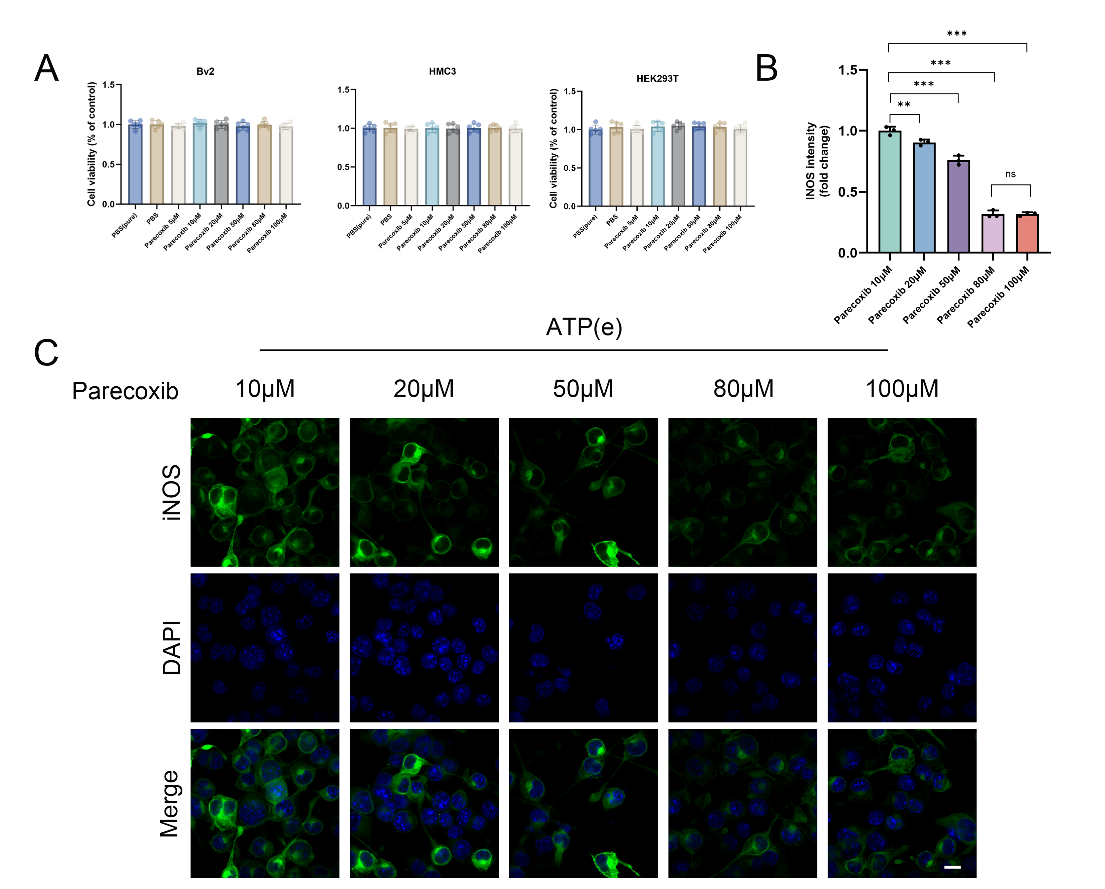


**Figure S4 Parecoxib's therapeutic impact on ATP-triggered microglial activation.** (A) The MTT assay was employed to assess the cytotoxic effects of parecoxib at concentrations of 5µM, 10 µM, 20µM, 50µM, 80µM and 100 µM on BV2, HMC3 and H293T cell lines. Parecoxib was solubilized in phosphate-buffered saline (PBS) for the experiments; (B, C) An immunofluorescence assay was employed to assess the expression of inducible nitric oxide synthase (iNOS) following a 24-hour co-treatment with extracellular ATP and varying concentrations of parecoxib. The plecoxib concentrations utilized were 10 µM, 20µM, 50µM, 80µM and 100 µM, with iNOS visualized in green and DAPI-stained nuclei in blue. *, **, *** denote p<0.05, 0.01, 0.001, Values are means ± S.E.M, Comparisons between two groups used two-tailed Student’s t-test; multi-group comparisons used one-way ANOVA.


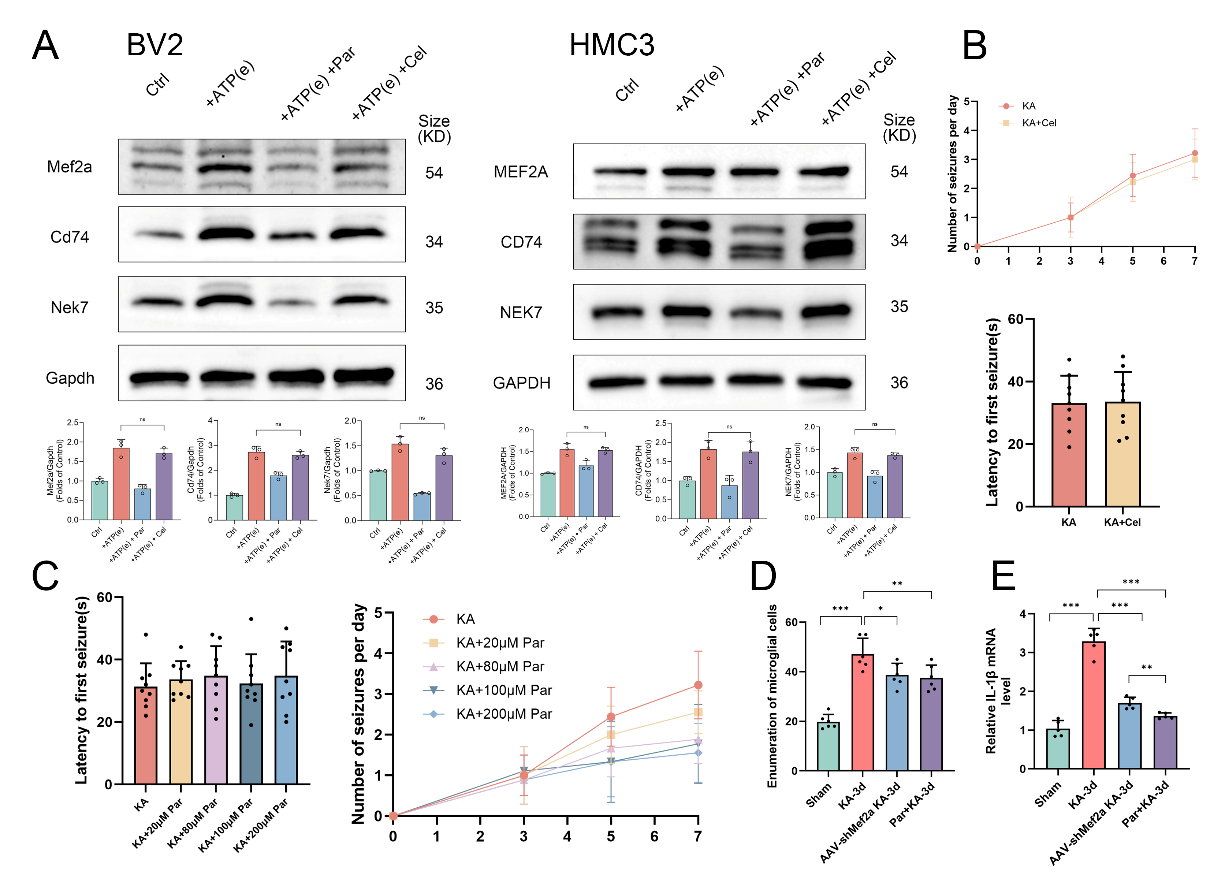


**Figure S5 Investigation of Parecoxib Concentration in Animal Studies.** (A) Western blot analysis of MEF2A, CD74, NEK7, and GAPDH protein expression in BV2 cells and HMC3 cells under control, ATP(e) treatment, ATP(e)+parecoxib and ATP(e)+ celecoxib treatment conditions. Quantified protein expression data analyzed by Student's t-test; n=3 per group. (B) Seizure latency and daily seizure frequency in KA and celecoxib+ KA groups; analyzed by Student's t-test, n=8 per group, p > 0.05. (C) This study investigates the effects of different concentrations of parecoxib (20 µM, 80 µM, 100 µM, and 200 µM) on the latency to the onset of first seizures and the daily seizure frequency in a kainic acid (KA)-induced epilepsy mouse model. For the experimental procedures, parecoxib was dissolved in phosphate-buffered saline (PBS). Each mouse received a stereotactic injection of 2 µL of parecoxib into the lateral ventricle, n=8 per group; (D) Quantification of microglial cell numbers in the hippocampal CA3 region from Sham, KA-3d, AAV-shMef2a KA-3d, and Par+KA-3d groups; analyzed by one-way ANOVA; n = 6 per group; (E) The expression level of IL1β mRNA in the hippocampal region of mice following administration of parecoxib and AAV treatment was quantified using quantitative polymerase chain reaction (qPCR). *, **, *** denote p<0.05, 0.01, 0.001, Values are means ± SD, Comparisons between two groups used two-tailed Student’s t-test; multi-group comparisons used one-way ANOVA.


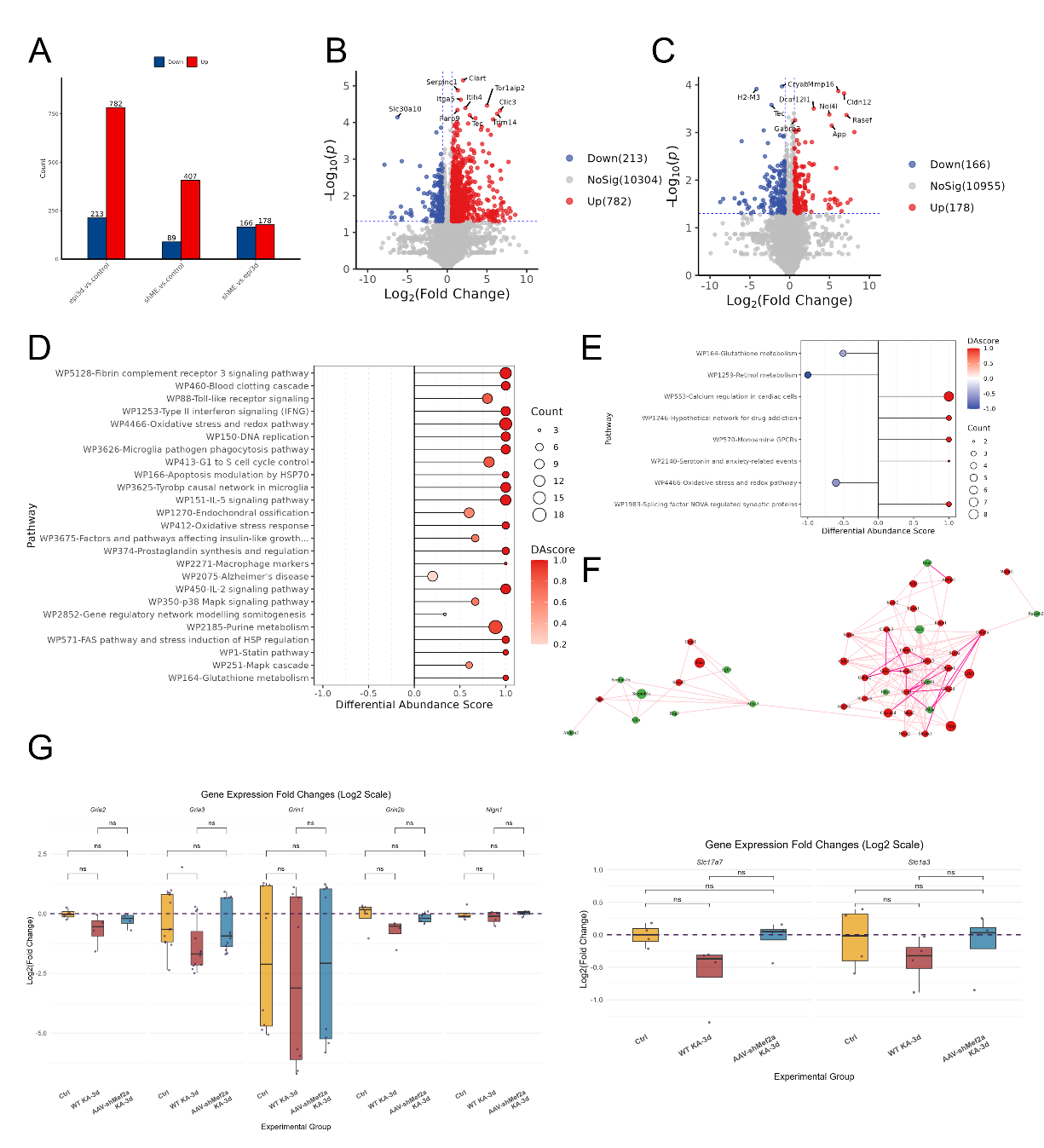


**Figure S6: Proteomic profiling elucidates differential protein expression and pathway modifications resulting from Mef2a knockdown.**  (A) Overview of differentially expressed proteins (DEPs) among the experimental groups. The bar graph illustrates the number of DEPs identified (criteria: fold change > 1.5, p < 0.05) in comparisons between Control (Ctrl), KA-3d (epi), and AAV-shMef2a KA-3d (shME) groups. (B–C) Volcano plots depicting global changes in protein expression between (B) Ctrl vs. KA-3d and (C) shME vs. KA-3d groups. Red and blue dots indicate proteins that are significantly upregulated (FC ≥ 1.5) and downregulated (FC ≤ -1.5), respectively (p < 0.05). (D–E) WikiPathway enrichment analysis based on Differential Abundance (DA) Scores for comparisons of (D) Ctrl vs. KA-3d and (E) shME vs. KA-3d. The top 30 significantly enriched pathways are presented. The DA Score (x-axis) ranges from -1 (indicating downregulation) to 1 (indicating upregulation). Dot size reflects the number of proteins per pathway, and the color gradient represents the magnitude of the DA score. (F) The Protein-Protein Interaction (PPI) network of differentially expressed proteins (DEPs) between the shME and KA-3d groups was constructed utilizing the STRING database (version 11.5) with a confidence score threshold greater than 0.7. In this network, nodes represent proteins, while edges indicate interactions between them. Key hubs are emphasized through variations in node size and connectivity. (G) A boxplot analysis was conducted on key proteins and transporters associated with excitatory synapses, including Gria2, Gria3, Grin1, Grin2b, Nlgn1, Slc17a7, and Slc1a3. The analysis revealed no statistically significant differences among the Ctrl, KA-3d, and shME groups, as determined by one-way ANOVA.


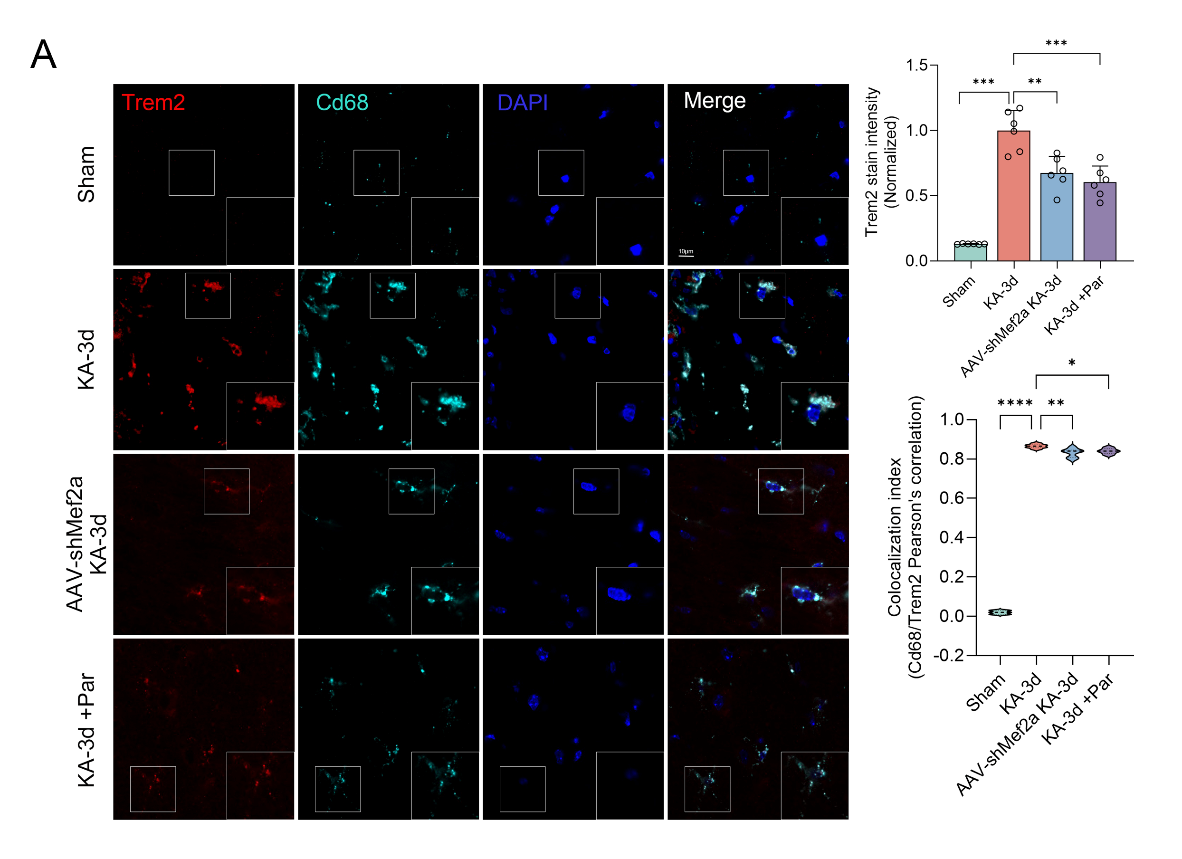


**Figure S7** (A) Dual immunofluorescence staining for Trem2 (red) and CD68 (light blue) in hippocampal sections from Sham, KA-3d, AAV-shMef2a KA-3d, and Par+KA-3d groups; scale bar = 20 µm (n = 6 per group). Quantification of GABAAR expression intensity and Pearson’s correlation coefficient for Trem2/ CD68 co-localization; analyzed by one-way ANOVA; statistical comparisons between Sham and treatment groups. Data are presented as mean ± SD from each independent experiment unless stated otherwise. Statistical comparisons were performed using one-way ANOVA. Significance levels: *p < 0.05, **p < 0.01, ***p < 0.001 vs. indicated Sham or comparison groups.
