# Supplementary figures and images for: Targeting MEF2A suppresses microglial hyperactivation and synaptic phagocytosis to attenuate epilepsy pathogenesis
Source: Cell Death Dis. 2026 May 22;17(1):645. doi: 10.1038/s41419-026-08860-5 (PMC13377041; doi:10.1038/s41419-026-08860-5)

Un-cropped images of the original western blots


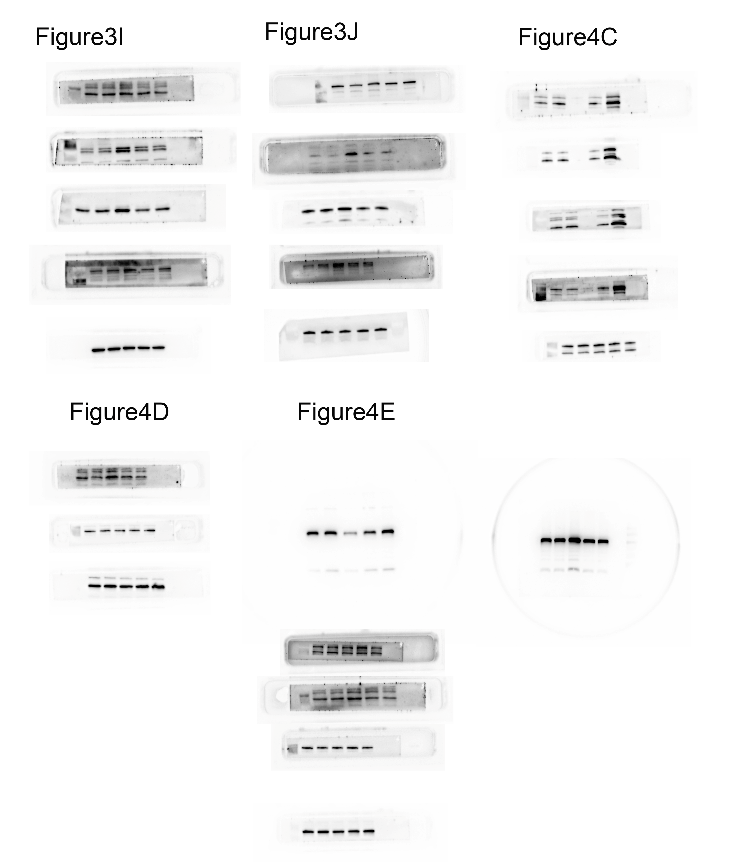


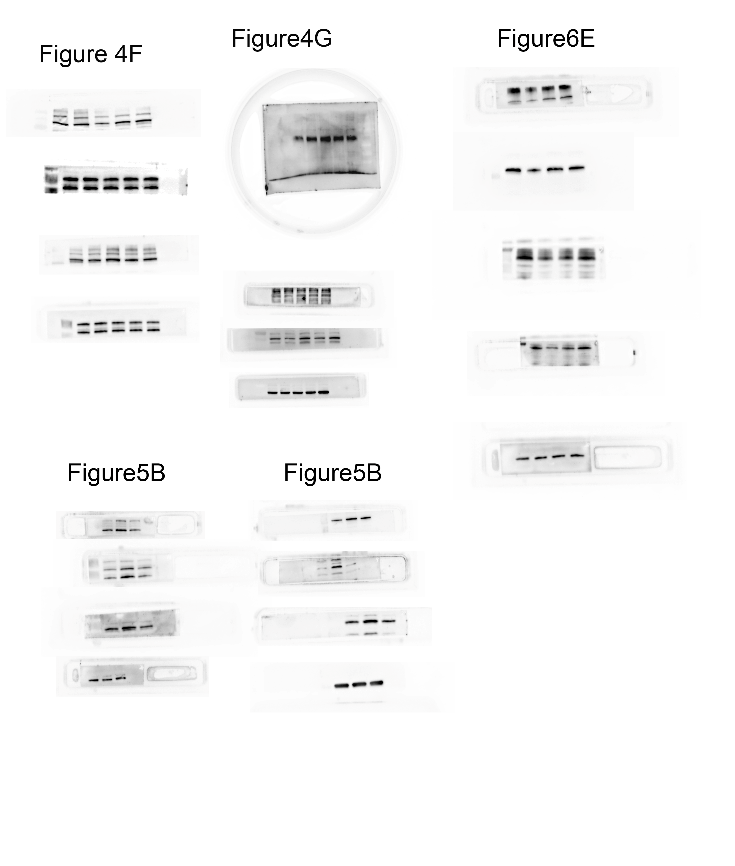


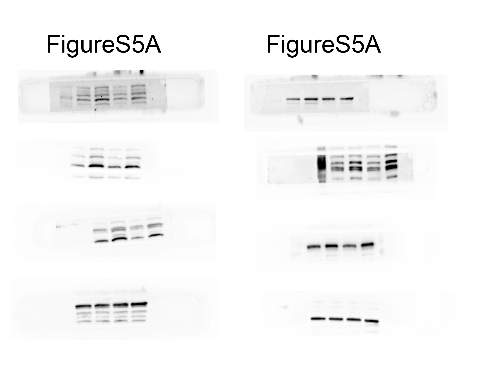

Supplement: Supplementary file 2 — Original Western Blots. [file 41419_2026_8860_MOESM2_ESM.docx]
